# Struggling, Forgotten, and Under Pressure: A Scoping Review of Experiences of Sex Workers During the COVID-19 Pandemic

**DOI:** 10.1007/s10508-023-02633-3

**Published:** 2023-06-13

**Authors:** Samantha K. Brooks, Sonny S. Patel, Neil Greenberg

**Affiliations:** 1 Department of Psychological Medicine, King’s College London, Weston Education Centre, London, SE5 9RJ UK; 2 Transcultural Conflict and Violence Initiative, Georgia State University, Atlanta, GA USA; 3 Department of Global Health and Population, Harvard T.H. Chan School of Public Health, Boston, MA USA

**Keywords:** COVID-19, Pandemic, Sex work, Sex workers, Well-being

## Abstract

The COVID-19 pandemic profoundly affected physical, mental, and economic well-being across the globe and has disproportionately affected certain vulnerable groups. This paper provides a scoping review of literature on the impact of the COVID-19 pandemic on sex workers, published between December 2019 and December 2022. Six databases were systematically searched, identifying 1009 citations; 63 studies were included in the review. Thematic analysis revealed eight main themes: financial issues; exposure to harm; alternate ways of working; COVID-19 knowledge, protective behaviors, fear, and risk; well-being, mental health, and coping; access to support; access to health care; and the impact of COVID-19 on research with sex workers. COVID-associated restrictions led to reduced work and income, leaving many sex workers struggling to cover basic needs; additionally, government protections excluded those working in the informal economy. Fearing the loss of their already reduced number of clients, many felt compelled to compromise both prices and protective measures. Although some engaged in online sex work, this raised concerns about visibility and was impossible for those without technological access or skills. Many feared COVID-19, but felt pressure to continue working, often with clients who refused to wear masks or share exposure history. Other negative impacts on well-being related to the pandemic included reduced access to financial support or health care. Marginalized populations (and especially those in professions which require close contact like sex workers) need further support and capacity-building within the community to recover from the impact of COVID-19.

## Introduction

Novel coronavirus 2019 (COVID-19) was declared to be a global pandemic in March 2020 (World Health Organization, 2020). As of January 2023, there have been over 671,000,000 cases of COVID-19 globally and over 6.7 million deaths (Worldometers, 2023). The pandemic also led to a global economic recession (House of Commons, 2021) and the strategies used to reduce spread of the virus (i.e., quarantine and lockdown) have had a major psychological burden on the global population (Bonati et al., 2022; Leung et al., 2022).

The pandemic has had far-reaching impacts across the world, affecting every population group in every society. However, there are some groups who are likely to have been disproportionately affected: vulnerable populations who already face structural disparities (e.g., disparities in terms of access to health care and support) tend to suffer the most during a crisis (Carter, 2021).

Individuals involved in sex work (that is, the exchange of sexual services for money or goods; Open Society Foundations, 2019) constitute one such vulnerable group. A report published by Fondation Scelles (2012) estimates that there are approximately 40–42 million sex workers across the world. COVID-19 has impacted all workers (Organisation for Economic Co-operation & Development, 2020), but there are expected to be particularly severe impacts on those with precarious contracts, including sex workers (Matilla-Santander et al., 2021).

Across the globe, sex work remains mostly illegal or “limitedly legal” (e.g., selling sexual services may be legal but soliciting, brothel-keeping, and buying sex may be illegal) (ProCon, 2018) and therefore workers may face stigma and difficulties accessing support. Indeed, in the early days of COVID-19 when many governments across the world introduced financial support packages to help citizens cope with the economic impact of the pandemic, sex workers were frequently excluded from accessing this support (Rana, 2020).

Additionally, while sex workers themselves are a marginalized group, many within this population also belong to other marginalized or vulnerable groups. For instance, many are migrants and/or ethnic minorities (Goldenberg et al., 2017; Platt et al., 2022) who take on work (including sex work) in the informal sector due to structural inequities including economic marginalization and racialization, unequal access to education and housing, and discriminatory immigration policies which increase vulnerabilities (Goldenberg et al., 2017; Platform for International Cooperation on Undocumented Migrants, 2019). There are also reported to be a disproportionate number of LGBTQ + sex workers (Fitzgerald et al., 2015; Platt et al., 2022). Sex workers from both sexual and gender minority groups and ethnic minority groups have been reported to experience more intensive police enforcement and more violence from police and clients (Platt et al., 2022). Sex workers also often live in areas of poverty and high unemployment rates (Benoit et al., 2015; Fielding-Miller et al., 2014), which are perhaps drivers of becoming involved with sex work in the first place (Fielding-Miller et al., 2014). Many have experienced homelessness (Macon & Tai, 2022), incarceration (Socías et al., 2015), mental illness (Beattie et al., 2020), neglect or abuse (Benoit et al., 2015); intimate partner violence (Hong et al., 2022), workplace violence (Deering et al., 2014) or substance use (Iversen et al., 2021). There is also a disproportionate burden of HIV within the sex worker community (Paz-Bailey et al., 2016), potentially related to economic vulnerabilities which affect sex workers’ ability to negotiate condom use (Gil et al., 2021). Additionally, the criminalization of sex work, police harassment, perceived stigma surrounding sex work and systemic racism are all reported to be structural barriers impeding sex workers’ access to health care (Goldenberg et al., 2022; Potter et al., 2022). Overall, the many risks that sex workers are disproportionately vulnerable to are compounded by the criminalization and stigma associated with sex work (Hail-Jares et al., 2015; Potter et al., 2022). This is certainly not to say that all sex workers also belong to other vulnerable groups and it is not our intent to construct sex workers as ‘victims’ as many reject that label (Jackson, 2016). However, it is important to acknowledge that the population of sex workers tends to disproportionately include individuals from other vulnerable populations. The sex worker population may therefore have been disproportionately negatively affected by the consequences of the COVID-19 pandemic.

Early in the pandemic, sex worker organizations, activists and academics highlighted the importance of protecting the rights of sex workers during the pandemic and raised concerns that the pandemic’s impact on this already marginalized group could lead to increased poverty, homelessness and severe health risks (Adebisi et al., 2020; Howard, 2020; Jacobson et al., 2020; Janyam et al., 2020; Platt et al., 2020). A joint statement by the Global Network of Sex Projects and UNAIDS called on governments across the world to take immediate action to protect the human rights of sex workers during the pandemic, ensure they were included in the allocation of financial support, and ensure the provision of adequate and non-judgmental health services to sex workers (UNAIDS, 2020) in order to ensure sex workers were not ‘left behind’ in the pandemic. They pointed out that health crises such as COVID-19 expose existing inequalities and disproportionately affect people who are already marginalized, drawing attention to the hardships facing sex workers (such as loss of income and increased discrimination and harassment). Numerous studies have been published exploring sex workers’ COVID-19 experiences. However, to date there has been no in-depth synthesis or review of the literature. Therefore, the aim of this study was to explore the literature relating to COVID-19 and sex workers, in order to synthesize findings and identify any gaps in the literature.

## Method

We followed Arksey and O’Malley’s (2005) scoping review framework, consisting of five stages: identifying the research question, identifying relevant studies, selecting studies for inclusion, charting the data, and summarizing the results.

### Identification of Research Question

As this was a scoping review, we opted for a very broad question in order to assess the full landscape of recent research with sex workers. The research question was, simply: What is known about the impact of the COVID-19 pandemic on sex workers? This enabled us to explore all different aspects of sex workers’ pandemic experience.

### Identification of Relevant Studies

For this review, we designed a search strategy consisting of two search strings. Search string 1 included terms relating to sex work (such as sex work, brothel, and escort) and search string 2 included terms relating to the pandemic (such as COVID, coronavirus, and lockdown). The full search strategy is presented in Appendix 1. Searches were limited to literature published after December 2019, when the COVID-19 pandemic began. Searches were carried out on December 10, 2022, across six databases: Web of Science, Embase, Medline, PsycInfo, Global Health, and Scopus.

### Selection of Studies for Inclusion

To be included, studies had to report primary data on any COVID-19 experiences (e.g., health-related, work-related, finance-related) of individuals involved in sex work or other “transactional sex”—that is, sex exchanged for material support or other benefits, which may be more informal than “sex work”: Wamoyi et al. (2019) suggest that transactional sex does not necessarily involve an explicit acknowledgement of the exchange of sex, and may involve non-commercial relationships. Participants did not have to be sex workers themselves but could be, for example, staff of NGOs involved in the support of sex workers, or clients of sex workers.

Studies were included if they were published in academic journals (i.e., preprints and other forms of gray literature were not eligible for exclusion). However, we did not have any inclusion criteria relating to peer review status (i.e., research published as letters, rather than articles, in order for rapid dissemination were eligible for inclusion as it was assumed that they would at least have undergone editorial review). Lastly, to be included, studies had to be published in the English language as this is the language spoken by the reviewers.

All citations located by our search strategy were downloaded to EndNote reference management software (Thomson Reuters, New York) where duplicates were automatically removed. The first author screened all titles for relevance, excluding any which clearly were not relevant to the review. The first author then screened all abstracts, excluding any which clearly did not meet the inclusion criteria. The first author then located full texts of all remaining citations and read the papers in their entirety to determine whether or not they met the inclusion criteria. The second author carried out independent screening of 10% of studies to ensure reliability of the screening process. Any disparities between the first and second authors’ inclusions were discussed between the authors until they reached agreement.

### Charting Data

Data were extracted from each study onto a Microsoft Excel spreadsheet containing the following headings: authors; year; country; methodological design; number of participants; age and gender breakdown of participants; focus of the study; measures used; key results; and conclusions. Data were extracted by the first author and the second author carried out data extraction of 10% of studies in duplicate to ensure reliability; again, any disparities between the first and second authors’ extracted data were discussed between the authors until they reached agreement.

The “key results” column of the data extraction spreadsheet was imported to NVivo (QSR International, version 12) where thematic analysis (Braun & Clarke, 2006) was used to analyze the results of the studies. Data were coded with codes summarizing its content: for example, any results relating to stress or anxiety were coded as “mental health and well-being,” and any results relating to changes in income during the pandemic were coded as “finance.” Codes similar in content were grouped together and developed into themes. Close examination of the data within each theme, along with discussion between the authors, led to the defining and naming of themes resulting in a final master list of themes.

### Summarizing Data

Each of the themes identified is summarized narratively in the Results section, including a description of what each theme is about and discussion of the evidence within the themes.

## Results

**Fig. 1:**
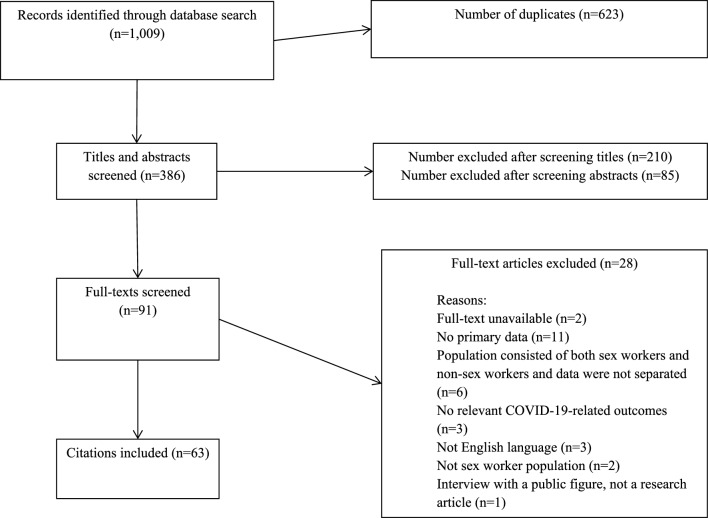
Screening process

Database searches yielded 1,009 citations, of which 623 were duplicates and immediately removed. Titles of the remaining 386 citations were screened, resulting in the removal of 210 citations which were clearly not relevant to the review. Abstract screening resulted in the removal of a further 85 studies, leaving 91 studies for full-text screening. Two full texts could not be found online and were therefore excluded. Twenty-six were also excluded for not meeting all inclusion criteria, which left 63 for inclusion in the review. A flow diagram illustrating the screening and selection process is presented in Fig. 1.

Included studies represented six continents, with Africa being the most represented (19 studies). Table 1 shows the countries in which participants of the included studies were based.

**Table 1:** Countries represented in included studies

Continent/country	Number of studies
*Africa*	*19*
Ethiopia	1
Ghana	1
Kenya	5
Mozambique	1
Nigeria	3
South Africa	2
Zimbabwe	6
*Asia*	*12*
India	4
Indonesia	2
Israel	1
Philippines	1
Singapore	2
Thailand	1
Vietnam	1
*Europe*	*11*
Germany	1
Italy	1
Netherlands	1
Poland	1
Portugal	2
Spain	3
UK	2
*North/Central America*	*7*
Canada	2
Dominican Republic	1
USA	4
*South America*	*3*
Brazil	3
*Oceania*	*1*
Australia	1
Multiple countries within one study	8
Not reported	2

The majority of studies included sex workers themselves as participants: 39 included sex workers, 4 included individuals involved in outreach or support for sex workers, and 16 included a combination of sex workers and individuals representing organizations which provided support for sex workers. One study included clients of sex workers and the remaining three involved content analysis of Web sites. Of those studies which included sex workers as participants, 29 included female sex workers only; 5 included male sex workers only; 13 included multiple genders and the remainder did not report the gender breakdown of participants.

The most common study design was qualitative (*n* = 23), mostly involving individual, semi-structured interviews. A further 11 studies used a combination of methods, with most of those also involving a qualitative element. 18 studies involved quantitative data, either cross-sectional or longitudinal. Other study designs included analysis of digital data/Web site content; case studies; mapping health data; and collaborative auto-ethnography.

The characteristics of the included studies are summarized in Table 2, and an overview of the themes emerging from the literature is presented in Table 3. A narrative summary of results is also presented in this section.

**Table 2:** Summary of included studies

Authors (year)	Country	Participants	Sociodemographics	Focus	Design	Outcomes assessed	Time period of data collection
Aantjes et al. (2023)	Mozambique	38 sex workers, 10 outreach workers, 7 informants with key positions in national COVID coordination bodies, Ministry of health and civil society organizations	Sex workers: 100% female Mean age not reported; range 18–24 Others: Not reported	The impacts of national COVID-19 restrictions on young adult sex workers in Mozambique and actions taken (at individual, governmental and civil society levels) to mitigate these	Qualitative interviews	Sex workers: Impact of COVID-19 on income, security, health and well-being; coping and support; health services utilization; COVID prevention and vaccine acceptability Key informants and outreach staff: National and institutional responses to the pandemic, insights into sex workers’ needs, mitigation strategies	Sex workers and outreach staff: February–April 2021 Key informants: June–October 2021
Al-Rawi and Zemenchik (2022)	Not reported (presumed global)	1458 (re)tweets from 22 sex worker Twitter accounts	68.2% cisgender women; 22.7% sexual and gender minorities (1/5 was transgender); 9.1% cisgender men	How public social media is used as a tool of professional and personal expression by sex workers during the pandemic	Qualitative content analysis of tweets	Content analysis of all tweets referring terms relating to the pandemic	December 2019–July 2021
Avwioro et al. (2021)	Nigeria	604 female commercial sex workers	100% female Age range 15–35; age-group with largest representation was 26–30 (33.1%)	COVID-19 knowledge, awareness and prevention practices	Cross-sectional survey	COVID-19 knowledge (e.g., of symptoms and prevention), awareness (e.g., exposure to COVID media) and preventive practices (e.g., use of face mask during sex); potential associated factors (marital status, age, alcohol/cigarette/drug use, average number of clients per day, years of sex work experience, support for government prohibition of commercial sex work)	May–July 2020
Azam et al. (2021)	Netherlands and Belgium	Not reported	Gender not reported Age-group most represented was 25–30 pre-lockdown, 20–25 during lockdown	Impact of the pandemic on prostitution markets	Analysis of Web sites with user-generated content about prostitution markets	Price, duration and type of services provided; sociodemographic characteristics of sex workers; textual comments and reviews	April 2019–April 2020
Benoit and Unsworth (2022)	Canada	10 staff members of sex worker organizations providing support, advocacy and education by, for and with sex workers	Not reported	Stigma and marginalization experienced during the pandemic	Qualitative interviews	Impact of the pandemic on sex workers; whether some groups of sex workers have been more affected than others; whether services have been offered; whether organization has introduced new services or modes of delivery	November 2020
Brouwers and Herrmann (2020)	UK	45 adult service Web sites (9 responded to questions); 7 sex worker-led organizations	N/A	The response of adult service Web sites to the pandemic	Mixed-methods	Public sections of Web sites were searched for pop-ups posts, banners, etc., about COVID-19 Web sites were asked 5 questions relating to whether services or features had been adjusted; whether advice had been provided regarding in-person services; whether contact with sex worker support organizations had been made; and any other actions taken in response to the pandemic Organizations were asked whether they had been contacted by or received donations from the Web sites; whether they knew of any further Web site responses to the pandemic; and the responses they would have liked to see	Web sites contacted 6 weeks after UK introduced lockdown measures; organizations contacted a further 4 weeks after that
Burgos and Del Pino (2021)	Spain	11 women involved in prostitution	100% female Mean age 32 (range 23–63)	The impact of confinement on the lives of sex workers	Qualitative interviews	Impact of confinement on their lives; fear of catching COVID-19; emotions and feelings; relationship with surroundings	April 2020
Cabras and Ingrasci (2022)	Italy	Interviews: 10 key informers including social workers, police officers, lawyers Observation: 6 outreach activities, including social workers, volunteers and sex workers	Not reported	The pandemic experiences of female migrant sex workers	Mixed-methods (interviews, participant observation)	Interview schedules not described. Observation included listening to and joining conversations between sex workers and social workers/volunteers	January 2020–January 2021
Callander et al. (2020a)	Global (North America 49.8%, Europe 23.8%, Asia 16.6%, Oceania 4.2%, South America 3.1%, Africa 1.6%)	19,388 online profiles of male sex workers (78,399 data points)	100% male Mean age 27.5 (range 18–80)	Effects of COVID-19 on male sex work	Longitudinal study of ecological digital data	Study assessed changes over time in online activity and qualitatively analyzed free-text sections of sex work profiles for any references to COVID-19	September 2019–May 2020
Callander et al. (2020b)*	Not reported—presumed to be global	N/A	Not reported	Longitudinal analysis of online sex work activity and content analysis of safer sex work guidelines	Longitudinal study of ecological digital data	Number of active, inactive and newly created profiles on a sex work Web site; number of profile views	May–August 2020
Callander et al. (2022)	USA	15 sex workers, 4 delivering services to sex workers, and 2 who were both	47% cisgender women, 29.4% cisgender men, 17.6% non-binary, 5.9% transgender women Median age 34 (range 23–54)	COVID-19 effects on the health and safety of sex workers in the USA	Qualitative interviews	Sex workers: How COVID-19 has affected sex work practices; service access; social/financial support; knowledge and awareness of COVID-19; self-reported mental health Support services: How COVID-19 has affected sex work; support and advocacy work; policy, social and financial support provided	May–August 2020
Chakrapani et al. (2022)	India	Survey: 132 men who have sex with men, including 57 (43%) engaged in sex work Interviews: 10 men who have sex with men, including 3 (30%) engaged in sex work	100% male Age for the subgroup of sex workers not reported	Economic well-being and healthcare access among men who have sex with men	Mixed-methods: Survey and interviews	Sexual stigma; internalized homonegativity; economic stress; stress due to social/physical distancing; depressive symptoms since lockdown; anxiety symptoms since lockdown; access to HIV and sexual and mental health services; COVID protective behaviors	April–June 2020
Chiang et al. (2022)	Brazil	219 women engaged in prostitution	100% female (78.8% cisgender, 21.1% transgender) Mean age 41.3 (range 19–73)	The exposure of prostitutes working in São Paulo to COVID-19	Cross-sectional survey	Work and behavior during pandemic; prevention and exposure factors to SARS-Cov-2; comorbidities; medication; close contact with individuals with COVID-19; symptoms or positive tests for COVID-19; hospitalization; post-COVID complications; vaccination status	May 2021
Couto et al. (2022)	Brazil	30 sex workers	100% female Mean age not reported; most (78.26%) aged between 18 and 35	Mental health, stressors and coping strategies of female sex workers during COVID-19	Qualitative interviews	Questions relating to how the pandemic has affected day-to-day life, mental health during the pandemic, and which care options had been taken as a result	September–October 2020
Cubides Kovacsics et al. (2023)	Netherlands	4 sex workers, 1 member of the support organization Spot 46	Not reported	(In)security of sex workers during COVID-19	Qualitative interviews	Questions relating to COVID-19	June–August 2020
Dziuban et al. (2021)	Poland	3 members of a sex worker-led activist and advocacy collective—academics, activists and/or sex workers	Not reported	The struggle of the sex workers’ community during the pandemic	Collaborative auto ethnography	N/A	December 2020
Fedorkó et al. (2022)	Austria, UK (England and Scotland), France, Germany, Greece, Ireland, Italy, Norway, Poland, Portugal, Spain and Switzerland	Members of 19 different organizations working with and providing support to sex workers, of which 14 were sex worker-led	Not reported	How the pandemic affected the socioeconomic, health and safety conditions of sex workers and how they responded to the first waves	Cross-sectional survey	Composition of the organizations and their sites; impact of COVID-19 on sex workers; type of response deployed by the organizations; collaboration with other organizations; key demands for improving conditions for sex workers during the pandemic	October 2020–February 2021
Folayan et al. (2022)	Nigeria	2076 total sample size of which 1056 (50.87%) engaged in transactional sex (i.e., entered into a sexual relationship with a man to get needed or important things such as food and clothing) and 921 (44.36%) who sell sex	100% women or girls (transgender or cisgender) Age of subgroups involved in sex work or transactional sex not reported	Factors associated with poor access to HIV, tuberculosis, and sexual and reproductive health services among women and girls with HIV	Cross-sectional survey	Impacts of COVID-19 on HIV, tuberculosis and sexual and reproductive health services access; economic and social situation; health and well-being	June–October 2021
Gbagbo (2020)	Ghana	35 commercial sex workers	85.7% female, 14.3% male Mean age not reported; age-group most represented was 29–39 (60%)	Experiences of sex workers during COVID-19 restrictions	Qualitative interviews	Perceptions and experiences since the imposition of restrictions on movement	April–May 2020
Gichuna et al. (2020)	Kenya	117 sex workers; 15 healthcare providers	Sex workers: 100% female Mean age not reported (range 16–46; more than half were under 33) Healthcare workers: Not reported	Access to health care during the pandemic	Qualitative interviews	Immediate experiences of coping under COVID-19 and lockdown including income; housing; health; service provision; food security; working conditions; doing sex work; police; gender-based violence	April–May 2020
Gonzalez and Garrido (2022)	Spain	2 activist migrant sex workers, 2 members of civil society entities with roles in pro-rights activism	Sex workers: 100% female Age not reported Civil society entities: Not reported	The usefulness of the concept of ‘deservingness’ of social benefits	Mixed-methods: Analysis of secondary sources, political discourse on social networks, and qualitative interviews	Not reported	Political discourse tracked from March–September 2020 Interviews March–September 2021
Hassan et al. (2023)	Kenya	117 sex workers; 15 healthcare practitioners	Sex workers: 100% female, age not reported Practitioners: Not reported	Challenges faced by sex workers living in urban informal settlements in Nairobi during the pandemic	Qualitative interviews	Sex workers: Experiences of working during the pandemic; challenges faced by sex workers Practitioners: Access to healthcare and practical solutions to the crisis for this population	May–June 2020
Jepson et al. (2022)	Australia	50 street-based sex workers (compared to 53 people with disability or their carers; 53 aged care workers; 54 refugee and asylum seekers; 61 deaf/hard of hearing; 50 Aboriginal and Torres Strait Islander people; 1121 general community)	Sex workers only: 90% female, 8% prefer not to say, 2% reported their gender was not listed Mean age not reported; most common age-group was 40–49 (42%)	How vulnerable communities seek and respond to COVID-19 public health information	Cross-sectional survey	Sources of exposure to COVID-19; opinions regarding trust in people/groups such as politicians and general practitioners; COVID-safe behaviors; and ease of finding COVID-19 information	May–July 2021
Jones et al. (2022)	Ethiopia	20 young people involved in commercial sex work	100% female Mean age not reported; 50% aged between 15 and 19, 50% aged between 20 and 24	Sexual and reproductive health in vulnerable youth	Qualitative interviews	Questions regarding experiences since the start of the pandemic, knowledge of COVID-19, experiences of the public health response impact on sexual and reproductive health and right to bodily integrity	June 2020
Judge and Jackson (2023)	USA	172 male sex workers providing services to men	100% male Mean age 32.14 for White men, 28.09 for Black men, 28.29 for men of other races (Latin, Mixed, Mediterranean, Arab, Asian, Native American or other)	Factors associated with advertising sex work services online during the pandemic	Longitudinal	Availability of men to carry out sex work, based on advertisements on a popular sex work Web site; age; race	Number of advertisements in March 2019, April 2020, January 2021 and February 2021
Kahambing (2021)*	Philippines	Massage therapists who welcome the possibility of sex work within their practice (‘n’ not reported) (It should be noted that they opted to be referred to as massage therapists, ‘sex worker’ was not their preferred identification	100% female Age not reported	Mental health of massage therapists who practice sex work during the pandemic	Not reported	Not reported	December 2020–June 2021
Kavanagh et al. (2021)	Kenya	1,725 women in rural Kenya (including 266 people engaged in sex work as a primary source of income and 904 people engaged in sex work as a secondary source of income)	100% female Mean age 29.3 (of whole sample; mean age for subgroup engaged in sex work not reported)	Economic security, food security, health and sexual behavior of women at high risk of HIV infection during COVID-19	Longitudinal surveys	Self-reported income, employment hours, number of sexual partners and transactional sex partners, food security, expected economic status in next 6 months, general health, health concerns, COVID-19 symptoms and prevention behaviors, access to health care	May–June 2020 (compared with data collected September 2019–March 2020)
Lahav-Raz et al. (2021)	Israel	33 workers of aid organizations working with individuals in the sex trade	Not reported	Challenges facing aid organizations working with sex workers during the pandemic	Qualitative interviews	Aid organization’s work during the pandemic; pandemic’s impact on clientele; challenges faced by the assisted population and aid workers	May–July 2020
Laikram and Pathak (2021)	Thailand	Interviews: 24 sex workers Case studies: 3 sex workers Focus group: 4 participants working for NGOs, 3 from local government agencies, 7 sex workers	Interviews: 8 female, 8 male, 8 third gender Age range for females 17–42; males 19–28; other gender 16–55 Case studies: 1 male, 1 female, 1 third gender Focus group: Not reported	Legal implications regarding the rights of sex workers during the pandemic	Mixed-methods: Qualitative interviews, focus group and case studies, as well as secondary data from government and NGO reports	Not reported	Not reported
Lamontagne et al. (2022)	Nigeria	4,556 females with or at risk of HIV including 1,733 (38.31%) who were sex workers and 1,994 (44.06%) who engaged in transactional sex	100% female Age of subgroup who were sex workers not reported	Effect of COVID-19 on food insecurity, financial vulnerability and housing insecurity among women and girls living with or at risk of HIV	Cross-sectional survey	Food insecurity; financial vulnerability; housing insecurity	May–September 2021
Leyva-Moral et al. (2023)	Spain	29 individuals who engaged in sex work	20 (69%) transgender women, 6 (20.7%) cisgender men, 2 (6.9%) who identified their gender as ‘queer’ and 1 (3.4%) non-binary	Impact of COVID-19 on sex workers in accessing health and social services	Qualitative interviews	Questions assessed knowledge and perception of COVID-19; experiences of sex work during lockdown; specific health and social needs; knowledge of available help resources; access to medical care; resources search strategies; barriers to accessing care; sex work experiences; discrimination in social/health services; experiences of violence	November 2020–February 2021
Machingura et al. (2021)*	Zimbabwe	Female sex workers attending HIV prevention and treatment services (‘n’ not reported)	100% female Age not reported	Risk of contracting HIV during COVID-19	Comparison of lockdown data with 2017 data	Client numbers; income; work conditions; condomless sex (compared to data from 2017)	April–October 2020
Magnani et al. (2022)	Indonesia	Female sex workers; ‘n’ not reported	100% female Age not reported	Impact of COVID-19 on HIV and AIDS control efforts among female sex workers	Analysis of data from local implementing units of a national HIV/AIDS program for sex workers	Number of sex workers continuing to work; number of entertainment areas open for business; number of clients; number of health facilities and mobile clinics continuing to offer HIV testing; monthly numbers of sex workers contacted via community outreach mechanisms; condoms distributed; HIV tests; 3-month retention rates for sex workers that had initiated ART	4 sources of monthly data between January–July 2020
Mantell et al. (2021)	Kenya	193 sex workers	100% female Median age 22; age range 18–24	Awareness and precautions relating to COVID-19 compared to HIV	Surveys (with participants of an ongoing randomized controlled trial beginning pre-COVID)	Questions relating to COVID-19 awareness and precautions, access to health services, sex work during Kenya’s state of emergency, impact of the pandemic (on income, access to food, clients, and violence from a client or main partner)	Initial study October 2019–February 2020; follow-ups between April–July 2020
Matambanadzo et al. (2021)	Zimbabwe	19,407 HIV-negative sex workers screened for PrEP	100% female Age not reported	Uptake of PrEP among sex workers during the pandemic	Mapping of clinical data	PrEP screening, initiation, follow-ups, refills and routine clinical follow-ups	Pre-lockdown (January–March 2020); during severe restrictions (April–June 2020); subsequent easing (July–September 2020) and during drug stock-outs (October–December 2020)
Mavhandu-Mudzusi and Moyo (2022)	Zimbabwe	10 sex workers	100% female Mean age not reported; all aged between 31 and 45, age-group most commonly represented 31–35	Experiences of sex work in the context of the COVID-19 pandemic	Qualitative interviews	Work experiences during the pandemic	December 2020–March 2021
Mlambo and Masuku (2022)	South Africa	11 commercial sex workers	100% female Age not reported	Social support for sex workers during the pandemic	Qualitative interviews	Interview schedule not reported; presumed to be questions about income and social support	Not reported
Moura et al. (2022)	Portugal	5 leaders of the support network created by the Movement of Sex Workers	Gender not reported Mean age not reported; range 28–46	The work of the Portuguese national Movement of Sex Workers and their perceptions of the impact of COVID-19	Longitudinal / qualitative interviews	The impact of COVID-19 on their work, sex work, and sex workers; what could improve response to these workers; second interview assessed continuity of work since the first interview	May–August 2020; participants interviewed 3 times each during this time
Moyo et al. (2022)	Zimbabwe	10 sex workers	100% female Mean age not reported; range 31–45	Utilization of HIV services during the pandemic	Qualitative interviews	Experiences utilizing HIV services during lockdown	December 2020–March 2021
Museva et al. (2021)	Zimbabwe	Surveys: 200 sex workers Interviews: Government officials, local private clinic manager, peer educators (‘n’ not reported)	Surveys: 88% female, 12% male Mean age not reported; age-group most represented was under 30 (55%) Interviews: Not reported	Impact of COVID-19 on sex workers’ livelihoods	Mixed-methods: Cross-sectional survey; qualitative interviews	Surveys: Income before and after lockdown; sustainability of adaptation strategies Interviews: challenges and opportunities encountered by sex workers due to COVID measures; coping and response strategies used by sex workers	April–June 2020
Nyabeze et al. (2022)	Zimbabwe	10 sex workers	100% female Age not reported	Resilience of female sex workers in the wake of COVID-19	Mixed-methods: Qualitative interviews, document analysis, naturalistic observation	Not reported	Not reported
Pearson et al. (2022)	Canada	208 female sex workers	100% female (11.5% transgender women) Median age 45 (range 36–52)	Barriers to accessing governmental income support for sex workers	Cross-sectional survey nested within an ongoing prospective cohort study	Pandemic impacts on housing and economic factors; work environment; safety, violence and policing; social outcomes; experience of intimate partner violence; social cohesion; access to COVID emergency income supports	April 2020–April 2021
Pereira (2021)	Portugal	13 sex workers	100% male Mean age 32.5 (range 23–47)	Motives, safe sex practices and social vulnerabilities in male sex workers during the pandemic	Cross-sectional survey	Motives for sex work, safe sex practices, impact of COVID-19 on personal and professional lives	January 2021
Pollard et al. (2021)	India	16 sex workers (along with 13 men who have sex with men and 15 transgender women)	Sex workers: 100% female 25% aged 20–29; 57% aged 30–39; 19% aged 40–19	HIV service delivery during the pandemic for vulnerable groups	Qualitative focus groups	HIV service access; risk behaviors; economic security; feedback to ensure service continuity	November–December 2020
Prior (2022)	Israel & Vietnam	10 men who paid women for sex (9 in Israel, 1 who had been traveling in Vietnam)	100% male Mean age 38.6 (range 22–53)	COVID-19 experiences of men who pay women for sex	Qualitative interviews	Considerations for paying for sex or abstaining from it; sex consumption patterns during the pandemic; online sex-for-pay; relationships with women who are paid for sex and men who pay for sex; perceptions of the impact of COVID on their experiences and the sex industry as a whole	April–July 2020
Rao et al. (2022)	South Africa	2776 sex workers newly initiating PrEP	100% cisgender females Mean age not reported; approximately 60% aged 25 or older	Persistence on oral PrEP among sex workers	Longitudinal	Use of PrEP—date of initiation; outcomes of subsequent monthly visits, including whether or not individuals stopped and restarted PrEP	September 2016–December 2020
Richtermain et al. (2022)*	Kenya	2090 women at risk for HIV enrolled; 1725 at first survey, 1731 at second survey during pandemic	100% female Median age 27	Trends in transactional sex during the first year of the pandemic	Longitudinal	Weekly income; employment hours; total number of sex partners and transactional sex partners in the past month; prices associated with transactional sex Early and later pandemic outcomes compared with those from 6 months pre-pandemic	Surveys every 6 months; participants enrolled between 2017 and 2018 and trial ended March 2020 Telephone surveys conducted between May and June 2020 and between November 2020 and February 2021
Rogers et al. (2021)	USA	46 street-based sex workers	50% cisgender male, 30.4% cisgender female, 13% transgender woman, 2.2% transgender man, 4.4% non-binary Mean age not reported; age-group most represented was 25–34 (51.1%)	Impact of the pandemic on health behaviors and social circumstances	Cross-sectional survey	Frequency of substance use and sexual behaviors in past 30 days and whether there were changes due to the pandemic; use of PPE; impact of COVID-19 on life including access to services/jobs or housing/food/other needs, changes in relationships, changes in mood or thinking	April–May 2020
Santos et al. (2021)	Global	2,732 men who have sex with men (including 274 (11%) who had ever engaged in sex work)	100% male Age of sex worker subgroup not reported	Effect of COVID-19 on economic situation, mental health, HIV prevention and HIV treatment in men who have sex with men	Cross-sectional survey	Impact of COVID on economic vulnerability; mental health; HIV prevention; testing and treatment and care impacts	April–May 2020
Santos et al. (2022)	Global—participants from over 150 countries	21,795 sexual and gender minority individuals, of whom 1456 (6.7%) had ever engaged in sex work	Sociodemographic characteristics of subgroup involved in sex work not reported	Disparities in COVID-19 impacts on HIV prevention and care among sexual and gender minorities	Cross-sectional	Impact of COVID on economic vulnerability; mental health status; HIV prevention, testing and treatment and care impacts	October–November 2020
Shankar et al. (2022)	India	2352 sex workers	2221 cisgender female; 63 transgender; 68 transgender male Age not reported	Stereotypes concerning the immobility of sex workers within informal labor markets, their indebtedness and bondage to informal creditors	Cross-sectional survey	Labor history, income and expenditure; access to state assistance; accommodation and rents; savings and credit histories including how finances were managed pre- and post-COVID	August–November 2020
Shekhar (2023)	India	174 (120 commercial sex workers, 31 ‘informers, pimps, madams or brothel-keepers,’ 8 social workers, 11 researchers, 4 child welfare committee or shelter home superintendents	Gender not reported Mean age not reported but the majority belonged to the 26–35 age-group	Economic condition and related risks faced by commercial sex workers in India	Rapid interview assessments	Economic status; change in customers during lockdown; financial exploitation	April–May 2020
Silva and dos Santos Câmara (2020)	Portugal & Brazil	2 transvestites involved in sex work; Reflections from 2 researchers	Sex workers: Described as ‘transvestites,’ referred to with female pronouns. Mean age 41 (range 40–42)	Challenges of anthropological research with sex workers during the pandemic	Qualitative interviews with participants; reflections from researchers	How participants are facing the pandemic; how the researchers can carry out anthropological research in times of social distancing	Not reported
Singer et al. (2020)*	Not reported; authors in the USA	21 people engaged in sex work	52% cisgender women; 33% transgender or gender fluid; 10% cisgender men; 14% declined to answer Age not reported	Preventing COVID-19 in sex workers	Qualitative interviews	Barriers to accessing health care	Date not reported; interviews took place during Illinois’ first ‘shelter-in-place’ phase
Singer et al. (2022)	USA	16 Black sex workers	31.25% cisgender women; 25% cisgender men; 6.25% genderqueer or non-binary; 31.25% transgender women; 6.5% intersex female Mean age 30.8 (range 23–42)	Health-related needs and experiences of Black sex workers in greater Chicago	Qualitative interviews	Physical, sexual and emotional health needs; conceptualization and experiences of safety; impact of COVID-19	December 2020–April 2021
Stevens et al. (2021)	UK (England)	3 individuals representing sex workers—either sex workers themselves or ‘topic experts’ who provided support services to this group	Not reported	To identify the needs of various socially vulnerable groups during the first COVID-19 wave	Qualitative interviews	How the pandemic affected health, well-being and life circumstances	April–May 2020
Su and Valiquette (2022)	Brazil	12 transgender Venezuelan asylum seekers and undocumented migrants, 6 key informants who were sex workers, trans-activists or humanitarian/NGO staff (unclear how many were sex workers)	Not reported	Experiences of migration, informal labor and sex work in LGBTQI + people	Mixed-methods: surveys and interviews	How COVID-19 affected livelihood	Not reported; dates of those interviews quoted in the text appear to be September–November 2021
Tan et al. (2021aª)	Singapore	Interviews: 24 stakeholders from the sex work industry; Surveys: 171 sex workers	Interviews: Not reported Surveys: 62% cisgender female, 35.1% transgender female, 2.9% cisgender male Mean age 41	The impact of the pandemic on sex workers’ health and social needs	Mixed-methods (interviews and surveys)	Interviews: How COVID-19 impacted sex workers Survey: Extent to which sex workers experienced hardships (income, food security, sexual compromise, access to medical care, housing insecurity)	April–October 2020
Tan et al. (2021b)	Singapore	Interviews: 24 stakeholders from the sex work industry; Surveys: 171 sex workers Cyber ethnography: 8 Web sites	Interviews: Not reported Surveys: 62% cisgender female, 35.1% transgender female, 2.9% cisgender male Mean age 41 Cyber ethnography: N/A	Reorganization of sex markets as a result of COVID-19	Mixed-methods (interviews, surveys, and cyber ethnography)	Interviews: Organizational change; impact of the pandemic on the sex work industry and its stakeholders; recommendations for policymakers Survey: Days of work per week; average number of clients; venues used; proportion of clients as regulars; type of clients before COVID-19; food and housing insecurity; sexual compromise; access to health care; income before and after COVID-19 Cyber ethnography: Client characteristics; ways of advertising; marketing and placement strategies; Web site design; quantity and quality of posts from sex workers or clients; impact of COVID-19 on interactions; how Web sites mediated interactions between sex workers and clients	April–October 2020
Tran et al. (2022)	Vietnam	8 female sex workers with HIV	100% female Mean age 40.5	Impact of the pandemic on people with HIV who are members of vulnerable groups	Qualitative interviews	Questions on knowledge of COVID and perceived risk of COVID, effect of COVID on income, effect of COVID on mental health, effect of COVID on antiretroviral treatment	July 2020
Tümpel and Cardone (2022)	Germany	1 sex worker who also works as a sexual assistant	Female participant Specific age not reported; born in early 1980s	Reactions of self-employed people from various sectors to the constraints and requirements during the pandemic	Case study (compared to multiple case studies from other sectors) involving interviews, participant observation, shadowing	Reflections on the pandemic and doing business under the pandemic circumstances	18 months starting spring 2019
Wang et al. (2022)	Dominican Republic	187 female sex workers living with HIV	100% female Mean age 42.5	Impact of the pandemic on HIV care and treatment outcomes in sex workers	Cross-sectional survey	Impact of COVID-19 on financial situation, mental health, substance use, partner abuse, HIV care and treatment, and receipt of government benefits	August–December 2020
Wirawan et al. (2022)	Indonesia	951 sex workers	100% female Median age 26	Impact of the pandemic and changes taking place in the Indonesian female sex worker community, and predictors of these changes	Cross-sectional survey	Questions on impact of behavioral changes on client frequency; shift to online sex services; adoption of COVID preventive practices; changes to condom use; fear of COVID-19	September–October 2020

*Published as a research letter; may not be peer-reviewed

**Table 3:** Themes emerging from the literature

Theme	Sub-themes	Key points
Financial issues	Reduction in work and income Food insecurity Housing insecurity Economic need to resume work Financial survival strategies	Substantial reduction in work (and therefore income) due to physical distancing measures; curfews; closure of sex work venues; travel restrictions; fear of law enforcement and of catching COVID-19; and dwindling demand Loss of income led to food and housing insecurity Financial pressure to return to work due to these insecurities plus growing debts; supporting families; and paying for children’s education Survival strategies included borrowing money (thus increasing debts); relocating to cheaper neighborhoods (which were less safe); and finding alternate work (which was often difficult due to lack of education, qualifications and experience as well as stigma against sex workers) Transgender, Black, and older sex workers as well as those who lived in brothels and those with children appeared to be most vulnerable to financial insecurity
Exposure to harm	Reduced negotiation power with clients Aggression and violence	Clients took advantage of sex workers’ financial pressures to demand discounts. Increased competition and reduced demand also led some to reduce their prices Need for money and clients resulted in pressure to accept conditions or clients they would not usually accept (for example, risky behaviors / unprotected sex) Increased aggression, violence and discrimination from clients, partners, the public and police—which could not be reported as sex work is illegal in most places Sex workers reported brutality, harassment and bribery from police
Alternate ways of working	Different venues for in-person work Online sex work	Sex workers explored different venues for work such as brothels, visiting clients’ homes or bringing clients to their homes; those with no private space to bring clients were disadvantaged Many offered online sexual services Barriers to this included fear of visibility and the lack of anonymity associated with the Internet; lack of adequate mobile data; lack of access to or skills relating to technology Online sex work was not seen as sustainable due to the pay not being good and sites regularly being removed
COVID-19 knowledge, protective behaviors, fear and risk	Knowledge of COVID-19 Protective behaviors Fear and risk	Mixed findings relating to knowledge and awareness of COVID-19 among the sex worker population Barriers to knowledge included language barriers in accessing relevant information and limited technological access Even when knowledge was good, protective measures were not always taken Masks were frequently discussed within the literature but many reported their clients would refuse to wear them Many felt at risk and feared COVID-19 but felt they had to continue working Very little information is available about rates of COVID-19 infection among the sex worker community
Well-being, mental health and coping	Impact of COVID-19 on well-being and mental health Coping strategies	Sex workers reported worries and concerns; anxiety, depressive symptoms; loneliness; poor sleep; fear; uncertainty; and increased substance use Ways of coping included humor; positive thinking; hope; religious coping; and communicating with loved ones
Access to support	Governmental financial support Support from nonprofit organizations Views of outreach and support organizations Social support	Many could not access financial support from their governments Barriers to accessing financial support included migrant status; not having paid taxes; having no bank account; no documentable income; and language barriers. Many of these barriers are particularly relevant for migrant sex workers Sex workers were left feeling forgotten, excluded, invisible and abandoned Nonprofit organizations provided supplies and information Providers from nonprofit organizations reported barriers including lack of consistent funding; lack of personal protective equipment; and sex workers not reaching out due to fear of being reported for violating COVID-19 guidelines Being able to help could lead to feelings of empowerment for the providers but could also negatively affect self-worth if they were unable to provide as much help as they wanted Most sex workers had very little social support, with many living away from their families and avoiding friendships outside of work due to fear of stigmatization Support from other sex workers was often their only form of support, although there were also competition and tensions within the community due to reduced demand
Access to health care	Health care in general COVID-19 care Sexual and reproductive care HIV care	Reports of access to general health care were mixed. Barriers to accessing health care included not wanting to draw attention to their place of work or migrant status; fear of stigmatization; inability to travel to healthcare sites; lack of health insurance and fear of large bills for care; and not knowing where to access care Sex workers appeared to experience some difficulties obtaining COVID-19 tests and vaccines Experiences with accessing sexual and reproductive care were mixed but many reported reduced access to contraception Experiences with HIV care were also mixed; some studies found PrEP uptake improved during the pandemic whereas others reported problems with accessing PrEP, ART and HIV visits Barriers to HIV care included forfeiting clinic visits to work during the day; not wanting to disclose HIV status in order to enter healthcare facilities; concerns about confidentiality if receiving care at home; no authorization letters enabling travel to clinics; and no access to computers for telemedicine
Impact of COVID-19 on research with sex workers		Issues with research with sex workers during the pandemic include difficulties speaking on the telephone (due to lack of privacy, lack of data, or lack of access to a phone) and the fact that many had moved locations during the pandemic. Given how many sex workers who do take part in research studies describe feeling ‘invisible,’ it is important to note that only the most ‘visible’ will be taking part in the first place; there are likely many sex workers who do not take part in research studies who may have been affected even more extremely by the pandemic

### Financial Issues

The most prominent theme, reflected in almost all of the studies, related to the economic instability and financial difficulties participants experienced as a result of the loss of work caused by the pandemic and reduced demand for their services. The reduction in work led to reduced income which in turn led to difficulties paying for food or housing. As a result, many felt forced to return to work, borrow money or turn to non-sex work in order to survive.

#### Reduction in Work and Income

Participants across the studies reported that their income was negatively affected due to working less or, in some cases, being unable to work at all due to the COVID-19 restrictions put in place and the fact there was less demand for their services due to the pandemic.

Nearly all studies across the world reported on the reduction in work and income for individuals involved in sex work during the COVID-19 pandemic (Aantjes et al., 2023; Azam et al., 2021; Burgos & Del Pino, 2021; Cabras & Ingrasci, 2022; Callander et al., 2022; Chakrapani et al., 2022; Fedorkó et al., 2022; Gbagbo, 2020; Hassan et al., 2023; Judge & Jackson, 2023; Kahambing, 2021; Kavanagh et al., 2021; Laikram & Pathak, 2021; Leyva-Moral et al., 2023; Lamontagne et al., 2022; Machingura et al., 2021; Magnani et al., 2022; Mantell et al., 2021; Matambanadzo et al., 2021; Mavhandu-Mudzusi & Moyo, 2022; Mlambo & Masuku, 2022; Moura et al., 2022; Museva et al., 2021; Nyabeze et al., 2022; Pearson et al., 2022; Pereira, 2021; Pollard et al., 2021; Richterman et al., 2022; Santos et al., 2022; Shankar et al., 2022; Shekhar, 2023; Singer et al., 2020; Su & Valiquette, 2022; Tan et al., 2021a, 2021b; Tran et al., 2022; Wang et al., 2022; Wirawan et al., 2022).

In some studies, rates of transactional sex halved (e.g., in Kenya; Kavanagh et al., 2021), while in others sexual encounters completely halted (e.g., in Belgium and the Netherlands; Azam et al., 2021). The percentage of participants reporting fewer clients were generally high (e.g., 68% in an Indonesian study (Wirawan et al., 2022); 84% in a study from Kenya (Mantell et al., 2021); 90% in a study from Zimbabwe (Machingura et al., 2021)). In the latter study, the average number of monthly clients during the pandemic was lower than the average number of *weekly* clients pre-pandemic (Machingura et al., 2021).

The reduction in work was attributed to the physical distancing measures in place (Cabras & Ingrasci, 2022); nighttime curfews forcing sex workers to work earlier in the day when there was reduced demand (Cabras & Ingrasci, 2022; Mlambo & Masuku, 2022; Nyabeze et al., 2022); the closure of sex work venues such as bars, restaurants, hostels, hotels, licenses brothels, truck stops and entertainment venues (Callander et al., 2022; Hassan et al., 2023; Leyva-Moral et al., 2023; Machingura et al., 2021; Matambanadzo et al., 2021; Moura et al., 2022; Nyabeze et al., 2022; Shankar et al., 2022; Tan et al., 2021a, 2021b; Tran et al., 2022); restrictions on mobility (Hassan et al., 2023; Machingura et al., 2021; Matambanadzo et al., 2021; Museva et al., 2021; Tan et al., 2021a; Tran et al., 2022); the need to hide from law enforcement who were looking for people breaking lockdown rules (Aantjes et al., 2023); fears of catching COVID-19 if they continued working (Matambanadzo et al., 2021; Pollard et al., 2021); and dwindling demand for sex workers (Aantjes et al., 2023; Callander et al., 2022; Pollard et al., 2021) perhaps due to clients’ fear of catching COVID-19 (Hassan et al., 2023; Machingura et al., 2021; Mlambo & Masuku, 2022) or clients’ own loss of income (Hassan et al., 2023; Tran et al., 2022). One study from the Netherlands and Belgium examining trends in sex workers’ activity (Azam et al., 2021) found that activity declined most sharply after lockdown restrictions were officially imposed, not before, indicating limited precautionary behavior until it was mandatory. However, overall, reduced working—or in some cases complete cessation of sex work—appeared to occur both out of necessity (i.e., complying with public health orders) and out of (reluctant) personal choice (i.e., reducing transmission risks for themselves and their clients) (Callander et al., 2022).

The one study exploring the views of sex workers’ clients also described a substantial reduction in paying for sex during the pandemic (Prior, 2022). This study included mostly participants based in Israel and one who had been in Vietnam during the pandemic. The men in this study reported their main reasons for not partaking in paid sex was the fear of being infected and the unknown nature of COVID-19, coupled with uncertainty around the sex workers’ COVID-19 protective measures and fearing infecting other family members with health conditions (interestingly, none of the participants voiced concern about infecting the women who are paid for sex). Other barriers to paying for sex included impaired finances; job precarity; fear of being fined for violating lockdown; and not wanting their movement history revealed via ‘track and tracing’ and potentially jeopardizing their marriages and reputations.

Few studies examined factors associated with levels of work reduction. In a study from the Netherlands and Belgium, Azam et al. (2021) found the biggest reduction in work for those living and working with other sex workers (e.g., in brothels); those of South American or Eastern European ethnicity; those aged 30–50; and those living in the Netherlands compared to Belgium, possibly due to differences between the two groups in compliance with COVID-19 policies. In Indonesia, Wirawan et al. (2022) found that more severe income reduction was associated with living in areas with high tourism dependency. In Singapore, Tan et al. (2021a) found that greater income loss was reported by transgender sex workers and migrants/non-permanent residents. Gichuna et al. (2020) reported that sex workers in Kenya with young children were less likely to work during the pandemic as they feared exposing their infants to COVID-19. Judge and Jackson (2023), in an analysis of active online profiles of male sex workers in the USA, found that Black sex workers saw the largest decline in advertising their services online; the odds of a Black male sex worker’s profile remaining active during the pandemic were almost 90% lower than the odds for a White sex worker profile. They also found that, after adjusting for race effects, each additional year increase in the advertised age of the sex worker was associated with a 35% increase in the odds of the profile remaining active during the pandemic. This difference in advertising probabilities could reflect a complex interplay of social and economic factors.

#### Food Insecurity

Related to the loss of income, food insecurity during the pandemic was a recurrent theme across the global literature (Aantjes et al., 2023; Burgos & Del Pino, 2021; Callander et al., 2022; Gonzalez & Garrido, 2022; Hassan et al., 2023; Kavanagh et al., 2021; Lamontagne et al., 2022; Leyva-Moral et al., 2023; Mantell et al., 2021; Mavhandu-Mudzusi & Moyo, 2022; Moura et al., 2022; Moyo et al., 2022; Museva et al., 2021; Pearson et al., 2022; Pollard et al., 2021; Rogers et al., 2021; Shankar et al., 2022; Silva & dos Santos Câmara 2020; Tan et al., 2021a, 2021b; Wang et al., 2022). Kavanagh et al. (2021) found that participants in Kenya who relied on transactional sex for income were 5.3% more likely than those who did not to report difficulty obtaining food in the previous month and 18.3% more likely to report being worried their household would not have enough food. Lamontagne et al. (2022) also found that participants in Nigeria who engaged in sex work had over three times higher odds of food insecurity during the pandemic than those not engaging in sex work. Food insecurity was particularly marked for sex workers who lived with children or dependent siblings, who had to ration food or basic commodities (Aantjes et al., 2023—Mozambique), and for venue-based sex workers and transgender sex workers (Tan et al., 2021a—Singapore).

#### Housing Insecurity

Reduced work and subsequent reduced income left sex workers in many studies across the world facing housing insecurity (Benoit & Unsworth, 2022; Burgos & Del Pino, 2021; Cabras & Ingrasci, 2022; Callander et al., 2022; Dziuban et al., 2021; Gichuna et al., 2020; Hassan et al., 2023; Laikram & Pathak, 2021; Lamontagne et al., 2022; Leyva-Moral et al., 2023; Mavhandu-Mudzusi & Moyo, 2022; Moura et al., 2022; Pollard et al., 2021; Rogers et al., 2021; Tan et al., 2021a, 2021b; Tran et al., 2022). Housing was threatened not only by lost income but by the loss of access to motels and short-term rentals (Callander et al., 2022) suggesting the pandemic may have disproportionately affected those of low socioeconomic status who were in unstable housing. Gichuna et al. (2020) found that many of their participants in Kenya lived in informal settlements and lacked the security of tenure, risking forced eviction.

In a Nigerian study, Lamontagne et al. (2022) found that sex workers had significantly higher odds of reporting housing insecurity during the pandemic than the general population. Additionally, sex workers in Singapore who were transgender or non-permanent residents experienced increased housing insecurity (Tan et al., 2021a). As a result of housing insecurity, sex workers faced evictions or staying with abusive partners (Benoit & Unsworth, 2022—Canada), while others were forced to move in with clients as they could not afford to pay rent (Cabras & Ingrasci, 2022—Italy). Organizations developed to provided support in a homelessness crisis were not able to help without proof of income, and since sex work is not recognized as work in many countries this meant sex workers could not access their support (Dziuban et al., 2021—Poland).

#### Economic Need to Resume Work

Sex workers across almost all studies reported needing to resume sex work while lockdown restrictions were still in place due to financial pressures. In addition to struggling to pay for housing and food, many were already in debt and could not afford to lose more (Burgos & Del Pino, 2021); sex workers with children had additional costs associated with education and online schooling such as wi-fi, laptops and tablets to enable their children to learn remotely (Dziuban et al., 2021; Hassan et al., 2023; Laikram & Pathak, 2021; Shekhar, 2023); migrant workers often had families in their home countries they sent money to (Burgos & Del Pino, 2021; Gonzalez & Garrido, 2022; Kahambing, 2021); and migrant workers who traveled to their homes during lockdown and then returned had costs associated with quarantine on their arrival back to the countries they worked in (Dziuban et al., 2021). In a study from Thailand, Laikram and Pathak (2021) found that those who identified as “other” gender as opposed to cisgender male or female were the most affected economically, for a number of reasons—they typically experienced lower demand and lower pay even pre-pandemic; they faced tough competition among themselves; and they often had more expenses such as beauty surgery, make-up, and expensive attire and accessories.

Two studies reported that not only did sex workers feel forced to return to work but some individuals who were not sex workers pre-pandemic chose to begin sex work for the first time due to economic necessity (Chakrapani et al., 2022; Su & Valiquette, 2022); these studies were carried out in India and Brazil, respectively.

#### Financial Survival Strategies

One of the most common strategies for surviving the loss of income was borrowing money from others. Sex workers reported borrowing money from relatives (Aantjes et al., 2023; Callander et al., 2022; Shankar et al., 2022); partners (Callander et al., 2022); friends (Callander et al., 2022; Shankar et al., 2022); their regular clients (Aantjes et al., 2023; Chakrapani et al., 2022; Shankar et al., 2022); brothel-keepers (Shekhar, 2023); and in some cases, those that trafficked them (Cabras & Ingrasci, 2022). It should be noted that loans may not be just a result of COVID-19; a study from India described how even pre-pandemic, many sex workers were forced to borrow from informal and exploitative channels as they could not access formal channels of financial support and while there were new borrowings during the pandemic, previous loans would have also spilled over (Shankar et al., 2022).

Another strategy described in a study from Mozambique involved relocating to cheaper neighborhoods—which were less safe and thus increased the risk of harm (Aantjes et al., 2023). Many sex workers continued working or returned to work very soon after initial lockdowns in order to try to maintain an income. Analyses of trends in the number of active profiles on sex work Web sites (advertising in-person services) revealed that there was a substantial decrease in Web site traffic in the early lockdown period (Callander et al., 2020a) but this trend began to reverse by May 2020 (Callander et al., 2020b) suggesting sex workers were returning to work. However, those who did return to work faced reduced power to negotiate with clients and heightened risk of abuse, as will be discussed in the next section.

Some sex workers tried to turn to alternate (non-sex) work but this was frequently unsuccessful. Those who tried to start or revive small businesses often found their profit margins squeezed by market saturation, price inflation and strict policing of trading hours (Aantjes et al., 2023; Museva et al., 2021) as well as the stigmatization of sex workers (Museva et al., 2021), while others did not have the capital to even try to start a business (Hassan et al., 2023). Formal employment opportunities were scarce as many sex workers did not have the education, qualifications or experience needed for stable non-sex-related jobs (Aantjes et al., 2023; Callander et al., 2022; Su & Valiquette, 2022; Tan et al., 2021a; Tran et al., 2022) or were not able to find non-sex work in fields where they did have skills (Callander et al., 2022). Most of the sex workers in Tran et al.’s (2022) study from Vietnam reported feeling they were not capable of anything other than menial non-sex-related jobs, stating they did not have the capacity to learn new skills or good enough health to work manual non-sex jobs. Participants in Hassan et al.’s (2023) Kenyan study reported that they were discriminated against for being sex workers and did not get hired for cleaning jobs in their neighborhoods because of their sex worker status; similarly, Laikram and Pathak’s (2021) participants in Thailand reported loss of non-sex work employment opportunities due to discrimination from their communities.

In three studies from Zimbabwe, sex workers did take on non-sex work, such as selling small items like food, firewood, alcohol and cigarettes (Mavhandu-Mudzusi & Moyo, 2022; Museva et al., 2021; Nyabeze et al., 2022). Shankar et al. (2022) found that sex workers in India moved in and out of other labor markets more freely than presumed, with many sex workers engaged in other employment such as street vending, contractual labor or housekeeping pre-pandemic. In this study, while some participants who were sex workers alone did find alternate non-sex work during the pandemic, the majority did not, and those exclusively in sex work were therefore at higher economic risk.

### Exposure to Harm

As detailed in the previous theme, many sex workers felt pressured to return to work in order to survive as without income, they struggled to pay for food or housing and many took on debts. Many, therefore, accepted clients during the pandemic but due to the restrictions in place and low demand for their services, they found they had no power to negotiate prices, sexual practices or condom use with their clients and many engaged in risky practices for little money. Structural violence against sex workers was exacerbated during the pandemic, with participants across many studies reporting an increase in abuse, harassment, violence and discrimination—from partners, clients, the general public and police.

#### Reduced Negotiation Power with Clients

The reduced number of potential clients, and desperation for income, meant that sex workers had little negotiation power with their clients. Many clients were not willing to pay pre-COVID rates and used sex workers’ desperation for money to take advantage of them, demanding discounts (Aantjes et al., 2023; Burgos & Del Pino, 2021; Hassan et al., 2023; Mantell et al., 2021; Pereira, 2021) or even refusing to pay at all after receiving services (Chakrapani et al., 2022; Fedorkó et al., 2022; Museva et al., 2021; Pereira, 2021). In Mantell et al.’s (2021) Kenyan study, as many of 79.3% of participants reported lower payments from clients. Clients in Spain used threats of the police to get cheaper prices (Burgos & Del Pino, 2021). The shortage of clients also increased competition between sex workers, leading some in the USA to charge lower rates for their services (Callander et al., 2022) while the loss of income and subsequent food insecurity meant some sex workers in Zimbabwe were forced to exchange sex for food rather than money (Machingura et al., 2021). Conversely, some sex workers in Kenya reported using the opportunity to increase their fees (Richterman et al., 2022), and some in Ghana charged their customers extra for being in their rooms as they could not work on the streets (Gbagbo, 2020). Decreasing fees was more common among street-based sex workers, those of lower socioeconomic status, and those facing housing or food insecurity in a study from the USA (Callander et al., 2022).

The need for money and clients, coupled with competition with other sex workers for a reduced pool of clientele, meant that sex workers felt pressured to accept conditions or clients they previously avoided. For example, the closure of sex work venues and heavy police surveillance in hotspot areas pushed some workers to unfamiliar, unvetted areas where they had no protection against malicious people (Aantjes et al., 2023; Callander et al., 2022; Nyabeze et al., 2022). This safety risk was particularly pressing for street-based and female (both cisgender and transgender) sex workers (Callander et al., 2022—USA), as well as those new to sex work who may be in less of a position to refuse clients (Chakrapani et al., 2022—in India). Tan et al.’s (2021a) study from Singapore found that transgender sex workers were more likely to experience increased sexual compromise. The need for clients left sex workers feeling unable to vet new clients which left them feeling vulnerable to COVID-19 (Singer et al., 2020). Others felt forced to take part in potentially risky sexual practices they would not otherwise have taken part in. For example, many felt their ability to negotiate condom use was reduced, or felt pressured to forego condoms as they could charge more (Callander et al., 2022; Chakrapani et al., 2022; Fedorkó et al., 2022; Machingura et al., 2021; Matambanadzo et al., 2021; Mavhandu-Mudzusi & Moyo, 2022; Nyabeze et al., 2022; Pereira, 2021). Gbagbo’s (2020) participants in Ghana reported that COVID-19 was the only disease being feared by their clients, leading to laxity in protection against sexually transmitted infections. While this led to heightened concerns about HIV in a study from Zimbabwe (Matambanadzo et al., 2021), there was some evidence that the greater risk perception may have in turn increased openness to pre-exposure prophylaxis or PrEP (Matambanadzo et al., 2021). Additionally, another study from Zimbabwe by Machingura et al. (2021) concluded that the overall reduction in client numbers was so much greater than the increase in sex workers willing to have condomless sex that the risk of HIV and sexually transmitted infections may have decreased, rather than increased, in this population during the pandemic.

#### Aggression and Violence

Participants described poorer treatment from clients during the pandemic in terms of aggression and violence (Burgos & Del Pino, 2021; Fedorkó et al., 2022; Gbagbo, 2020; Hassan et al., 2023; Laikram & Pathak, 2021; Mantell et al., 2021; Su & Valiquette, 2022). Part of the reason for increased risk of violence correlated with the pressure for sex workers to meet clients at their homes (Hassan et al., 2023—Kenya); sex workers in Mozambique reported hearing stories of violent crimes and murders occurring in client homes (Aantjes et al., 2023). Due to the criminalization of sex work, sex workers in Germany were reluctant to come forward if assaulted (Tümpel & Cardone, 2022). Participants in a study from the Dominican Republic also reported an increase in verbal, emotional and physical abuse from partners (Wang et al., 2022). Some sex workers were forced to live in dangerous situations out of necessity; for example, Silva and dos Santos Câmara (2020) report that some transgender sex workers in Portugal and Brazil had no choice but to move back in with families who had forced them to leave after their gender identity was disclosed.

Sex workers also felt there was an increase in stigma and discrimination against sex workers during the pandemic (Mantell et al., 2021—Kenya) due to sex workers being perceived as carriers of COVID-19 (Gichuna et al., 2020—Kenya), and many experienced violence and harassment from security patrol teams and members of the public but felt unable to report anything to police because their work was illegal (Gbagbo, 2020; Laikram & Pathak, 2021; studies from Ghana and Thailand, respectively). Bullying was a particularly common experience for transgender sex workers in Thailand (Laikram & Pathak, 2021). Some reported that lockdown restrictions were imposed in a discriminatory way—for example, sex workers in the UK reported lockdown regulations particularly targeting migrant women and Black, Indigenous and people of color (Fedorkó et al., 2022). The same study revealed that in Norway sex workers had very low trust in law enforcement authorities because the regulations were reportedly used to arrest, fine and deport foreign sex workers; in both Norway and France, there were reportedly headlines depicting sex workers as COVID-19 spreaders.

In Mozambique, exposure to harm was worse for street-based sex workers than “elite” sex workers, defined as those who operated from luxury hotels and liaised with affluent clients (Aantjes et al., 2023). Street-based sex workers felt their visibility was increased due to COVID control measures (Callander et al., 2022) and as a result, many participants reported heightened exposure to bodily and mental harm from police (Aantjes et al., 2023; Chakrapani et al., 2022; Dziuban et al., 2021; Mavhandu-Mudzusi & Moyo, 2022; Mlambo & Masuku, 2022). Lockdown restrictions and curfews meant there was greater police activity, with police carrying out patrols of the streets and raids of clandestine bars and parties. If sex workers were found by police to be disobeying stay-at-home orders, they faced police harassment (Hassan et al., 2023; Mavhandu-Mudzusi & Moyo, 2022; Mlambo & Masuku, 2022); being removed from public spaces (Aantjes et al., 2023); bribery in the form of money or sex (Chakrapani et al., 2022; Hassan et al., 2023; Mavhandu-Mudzusi & Moyo, 2022; Mlambo & Masuku, 2022); large fines (Dziuban et al., 2021; Fedorkó et al., 2022); forced disclosure of sexual practices (Mavhandu-Mudzusi & Moyo, 2022); arrest (Mavhandu-Mudzusi & Moyo, 2022); and migrant sex workers faced deportation to their countries of origin (Aantjes et al., 2023). In some African countries (Mozambique, Kenya and South Africa) sex workers also reported facing police brutality (Aantjes et al., 2023; Hassan et al., 2023; Mlambo & Masuku, 2022). This exposure to harm and harassment from the police had a negative psychological impact on sex workers, causing them frustration, confusion and stress (Mavhandu-Mudzusi & Moyo, 2022).

These findings suggest that structural violence against sex workers was exacerbated during the COVID-associated restrictions. Mlambo and Masuku’s (2022) participants in South Africa reported feeling that the lack of activism and support for sex workers was exploited by the police to harass them, and participants called for new regulations of their industry to reduce police harassment.

### Alternate Ways of Working

Sex workers experienced many difficulties finding work in their usual ways during the pandemic, due to the many restrictions in place, social distancing, and reduced demand. This led many to seek different ways of working, such as meeting clients in non-public places, or using digital and social media to offer “online sex work.” However, there were numerous barriers to offering services online, including lack of technological skills, lack of private space to work at home, and lack of anonymity.

#### Different Venues for In-Person Work

The loss of income and reduction in clients resulted in the adoption of survival strategies such as changing methods of work. For example, some sex workers in Zimbabwe changed from street-based work to brothel-based (Mavhandu-Mudzusi & Moyo, 2022); participants in Mozambique reported meeting with clients at home or in guesthouses through advertisements on Facebook or WhatsApp sex groups (Aantjes et al., 2023). However, some were unable to have clients visit their homes—for example, those who shared accommodation with other sex workers or those with children at home—putting them at more of a disadvantage than those with adequate housing and private spaces to meet clients (Hassan et al., 2023; Moura et al., 2022).

#### Online Sex Work

Another way of increasing work opportunities involved deploying digital and social media skills to provide virtual sex services. A number of studies found an increase in online sex work during the pandemic (Al-Rawi & Zemenchik, 2022; Benoit & Unsworth, 2022; Cabras & Ingrasci, 2022; Callander et al., 2020a, 2022; Leyva-Moral et al., 2023; Mlambo & Masuku, 2022; Moura et al., 2022; Nyabeze et al., 2022; Rogers et al., 2021; Tan et al., 2021b; Wirawan et al., 2022). In a study from Indonesia, engaging in online sex work was associated with high fear of COVID-19 and adherence to sanitation and personal protective measures (Wirawan et al., 2022).

However, many were unable or reluctant to take part in online sex work. Barriers included not having private space to work from due to families being at home (Dziuban et al., 2021); fear around online content being public and ungovernable and making them more visible (Cabras & Ingrasci, 2022; Callander et al., 2022; Moura et al., 2022); lack of anonymity associated with the Internet (Al-Rawi & Zemenchik, 2022; Callander et al., 2022); fear of harassment through leaking of nude pictures (Nyabeze et al., 2022); lack of choice over clients (Cabras & Ingrasci, 2022); not having enough mobile data to provide services to their clients (Nyabeze et al., 2022); and lack of technological knowledge, access or skills (Callander et al., 2022; Mlambo & Masuku, 2022; Moura et al., 2022). This highlights how disparities in knowledge of how to use technology and access to computers or smartphones determine how COVID-19 was experienced (Mlambo & Masuku, 2022—South Africa). Finding sex work through virtual channels was also deemed harder for sex workers of color in the USA who felt they were already marginalized (Callander et al., 2022).

Online sex work was also deemed inadequate for various reasons. Firstly, the money made from online sex work was generally deemed to be only a fraction of what sex workers would be making on the streets (Callander et al., 2022; Moura et al., 2022). Secondly, participants in Singapore reported that online sites were often temporary as they got taken down by authorities (Tan et al., 2021b). Finally, sex workers felt online work was not sustainable due to lack of demand (Tan et al., 2021b).

An exploration from Singapore of the content of sex work Web sites themselves (Tan et al., 2021b) revealed that they were like a combination of shopping Web sites and personal ad sites, with sex workers being sold like “products” online: this could be demeaning for the workers. Stakeholders who were interviewed in the same study highlighted the importance of more research into virtual sex work as there may be different risks associated with this form of work (e.g., in terms of workers’ rights and the potential they will have to take part in activities they are not comfortable with). Additionally, in a study from the UK, Brouwers and Herrmann (2020) found that sex work Web sites appeared to show little responsibility toward those on their platforms, with most offering no financial support; continuing to charge for advertising; and many not displaying any acknowledgement of COVID-19 on their Web sites. Some Web sites did take measures to protect their users, including advice about COVID-19, new online features such as webcam or phone services, scrapping of advertising charges (only 20% of sites, *n* = 9), additional safety features such as signposting to support services, and donations to sex worker-led organizations (just 6.67%, *n* = 3). Participants in this study suggested that Web sites could help by providing free or reduced fee advertising, increased online opportunities, increased support and security for online features, donations to sex worker-led organizations, promoting such organizations to Web site users, direct engagements with such organizations, and urging clients (rather than workers) to avoid in-person meetings.

### COVID-19 Knowledge, Protective Behaviors, Fear, and Risk

Within this theme, we discuss participants’ understanding and perceptions of COVID-19. We found mixed results regarding sex workers’ knowledge of the virus, with language barriers and lack of Internet access often impeding their ability to access and understand the current guidance. It is therefore unsurprising that sex workers’ adherence to the COVID-19 guidelines was not always high. Many attempted to use protective measures but were not always unable to—for example, some clients refused to wear masks, relating to earlier findings around sex workers’ lack of negotiation power during the pandemic. Many feared COVID-19 and felt at risk, but the need for money often outweighed these fears.

#### Knowledge of COVID-19

Studies reported mixed findings on the depth of COVID-19 knowledge among sex workers. For example, Avwioro et al. (2021) found that knowledge and awareness in Nigeria were good overall, and Al-Rawi and Zemenchik’s (2022) analysis of global Twitter posts found that some sex workers used social media to share and promote messages aligning with government and public health recommendations to reduce COVID-19 spread. However, other studies reported poor knowledge. In Burgos and Del Pino’s (2021) Spanish study, despite reportedly being very afraid of catching COVID-19, participants were unclear about how to protect themselves. Dziuban et al. (2021) found that many migrant sex workers in Poland were not sure what the COVID-19 guidelines were or whether they were breaking them because their language skills for the countries they were working in were not sufficient to understand the restrictions. In an Australian study, Jepson et al. (2022) found that sex workers were significantly less likely than other groups to find current COVID-19 messaging relevant to them, but they were not more likely to find it harder to find or understand. Stevens et al.’s (2021) participants in the UK reported difficulties finding out about the rapidly changing COVID-19 government guidance and following the recommendations, and there were some cases of sex workers believing COVID-19 was a hoax and misidentifying COVID-19 symptoms as other infections or colds. In the same study, lack of or limited access to the Internet/technology was a barrier to accessing COVID-19 guidance.

An analysis of factors associated with good COVID-19 knowledge and awareness in a Nigerian study (Avwioro et al., 2021) found that good knowledge of COVID-19 was associated with use of alcohol, smoking or drugs; 2–4 years of sex work experience as opposed to less than a year; and older age. Awareness of COVID-19 management was significantly lower in single participants (as opposed to widowed or had children outside wedlock); younger participants; those who did not engage in alcohol, cigarette or drug use; participants who had sexual encounters with 1–2 or 5–6 persons per day as opposed to 3–4; participants with fewer years’ experience; and participants who supported government prohibition.

#### Protective Behaviors

Few studies explored sex workers’ adherence to COVID-19 guidelines/recommendations for reducing spread. Avwioro et al.’s (2021) Nigerian study found that despite knowledge and awareness being generally good, less than half (41.1%) implemented good practice regarding preventive measures. Wirawan et al. (2022) reported that approximately half of their sex worker participants in Indonesia had low adherence to sanitation and personal hygiene measures at work, and that adherence depended on the location of participants and their HIV status.

Wearing masks was the most commonly discussed preventive measure; findings regarding the wearing of masks were mixed. Cabras and Ingrasci (2022) reported that participants in Italy provided mixed views about masks, with some very concerned about COVID-19 and always wearing masks, and others denying the seriousness of the virus or accepting it as a risk of the job. Wirawan et al. (2022) found that 62% of participants in Indonesia adhered to use of personal protective equipment during sex work, and that adherence was associated with education. Chiang et al. (2022) found that 77% of participants in Brazil wore masks on the streets, but only 59% of participants always used masks during sessions; in a study by authors based in the USA, Singer et al. (2020) found that while some participants wore personal protective equipment when traveling to clients, none wore it during sex work. Mask-wearing was more common in Mantell et al.’s (2021) Kenyan study, with 80% wearing them. Jones et al.’s (2022) participants in Ethiopia reported it was easier to convince clients to use face masks than condoms, but participants in Gbagbo’s (2020) and Moura et al.’s (2022) studies (in Ghana and Portugal, respectively) reported that often clients would refuse to wear masks or would remove them, and Leyva-Moral et al.’s (2023) participants in Spain reported that clients would request the removal of masks and it was difficult to reject their requests. Additionally, a transgender sex worker in Laikram and Pathak’s (2021) study from Thailand reported that they were reluctant to wear facemasks as they were frequently abused by clients who felt they were concealing their true identity. This suggests that, again, there are disparities within the sex work community whereby those with additional vulnerabilities (e.g., transgender) may struggle more with adhering to COVID-19 guidelines.

Other protective measures taken included temperature checks (Callander et al., 2020a, 2022); COVID-19 testing (Callander et al., 2020a, 2022); frequent hand-washing (Gbagbo, 2020; Leyva-Moral et al., 2023; Mantell et al., 2021); asking clients if they had been around anyone with COVID-19 symptoms (Callander et al., 2022); hand sanitizer (Callander et al., 2020a; Gbagbo, 2020; Mantell et al., 2021); frequent showers (Callander et al., 2022); ventilation (Leyva-Moral et al., 2023); disinfectant (Pereira, 2021); staying at home more (Mantell et al., 2021); and longer gaps between clients (Callander et al., 2022). Despite protecting themselves to the best of their abilities, overall sex workers relied on the willingness of their clients to agree to protective measures (Burgos & Del Pino, 2021).

Sex workers in Spain reported being “hidden away” by their managers/the people who exploited them if they showed COVID-19 symptoms, but this was not so much to reduce the risk of spreading the virus as to avoid clients seeing the symptomatic workers and losing their business (Burgos & Del Pino, 2021).

#### Fear and Risk

Some sex workers acknowledged that their work put them at a greater risk of catching COVID-19 (Singer et al., 2020), while others felt at no more risk than anyone else (Moura et al., 2022). Many sex workers in Spain described being at heightened risk of catching COVID-19 due to their living arrangements if they lived and worked in brothels, sometimes in apartments with up to ten women in overcrowded rooms (Burgos & Del Pino, 2021). They were also at risk due to contact with clients, whose health status was often unknown (Burgos & Del Pino, 2021; Cabras & Ingrasci, 2022; Cubides Kovacsics et al., 2023; Moura et al., 2022). Tan et al. (2021a) found that sex workers in Singapore were afraid of catching COVID-19 at work, but felt forced to work anyway due to their inability to access financial support; putting themselves at risk of COVID-19 was perceived to negatively affect their mental health. Rogers et al. (2021) also found that participants in the USA reported being very fearful of COVID-19. In the two studies which quantitatively assessed fear of COVID-19, approximately half of the participants were fearful: 52.9% in Kenya (Mantell et al., 2021) and 48.2% in Indonesia (Wirawan et al., 2022) reported being concerned or fearful about catching the virus. In Wirawan et al.’s (2022) study, higher fear of COVID-19 was associated with income reduction, taking up online sex services, use of personal protective equipment at work and adherence to sanitation/hygiene measures. In Portugal, sex workers with chronic diseases were also fearful of catching COVID-19 and therefore less likely to return to work (Moura et al., 2022).

In Moura et al.’s (2022) Portuguese study, most sex workers reported not knowing of any COVID-19 cases; those that did know people who tested positive did not believe it was related to their sex work. Only one study reported the number of sex workers who had ever actually tested positive for COVID-19. Chiang et al. (2022) found that 11.7% of their participants in Brazil had tested positive, although over half reported symptoms; potentially, lack of access to testing may have skewed the results. Indeed, most of the participants in Singer et al.’s (2020) study had never been tested for COVID-19 due to concerns about the expense of the tests.

### Well-Being, Mental Health and Coping

A substantial amount of literature explored the psychological well-being of sex workers during the pandemic. Within this theme, we found evidence that COVID-19 lockdown led to many negative emotional states and exacerbated existing mental health problems. More positively, participants also named a number of coping strategies which helped them during the pandemic, including use of humor, positive thinking, hope, religiosity, and communicating with loved ones.

#### Impact of COVID-19 on Well-Being and Mental Health

In a content analysis of sex workers’ posts on Twitter (Al-Rawi & Zemenchik, 2022), “concern” was the most frequently occurring theme, with sex workers expressing worries about loved ones, health issues, essential workers, intersectionality, the criminalization of sex work and online safety.

Sex workers described how the pandemic negatively affected their well-being, reporting that they struggled with feeling anxious (Burgos & Del Pino, 2021; Couto et al., 2022; Lahav-Raz et al., 2021; Leyva-Moral et al., 2023; Mavhandu-Mudzusi & Moyo, 2022; Rogers et al., 2021; Wang et al., 2022); depression (Laikram & Pathak, 2021; Leyva-Moral et al., 2023; Rogers et al., 2021; Wang et al., 2022); emotional flooding (Lahav-Raz et al., 2021); sadness (Burgos & Del Pino, 2021; Leyva-Moral et al., 2023); difficulties sleeping (Couto et al., 2022; Lahav-Raz et al., 2021); fear (Couto et al., 2022; Lahav-Raz et al., 2021; Leyva-Moral et al., 2023); loneliness (Burgos & Del Pino, 2021; Lahav-Raz et al., 2021; Leyva-Moral et al., 2023; Wang et al., 2022); irritability (Couto et al., 2022); aggression (Lahav-Raz et al., 2021); post-traumatic symptoms (Lahav-Raz et al., 2021); flashbacks (Lahav-Raz et al., 2021); anguish and despair (Burgos & Del Pino, 2021; Leyva-Moral et al., 2023); psychotic symptoms such as delusions and paranoia (Rogers et al., 2021); and uncertainty over the future (Couto et al., 2022; Leyva-Moral et al., 2023; Mavhandu-Mudzusi & Moyo, 2022).

Participants also reported that the pandemic exacerbated existing mental health problems (Callander et al., 2022) and negatively affected the use of substances such as alcohol and drugs (Callander et al., 2022; Couto et al., 2022; Lahav-Raz et al., 2021; Nyabeze et al., 2022; Wang et al., 2022). However, one study from the USA (Rogers et al., 2021) found that while many reported increased substance use, an equal number reported decreased substance use.

Poor well-being was attributed to a combination of financial pressure and little social contact with others (Burgos & Del Pino, 2021—Spain) as well as fears of infection and distress resulting from the behavioral changes imposed by lockdown measures (Pereira, 2021—Portugal). A global study examining psychological distress in vulnerable groups found that members of vulnerable groups, including sex workers, suffered more severe psychological distress during the pandemic than groups who were not considered vulnerable (Santos et al., 2022). In the Dominican Republic, Wang et al. (2022) found that mental health challenges were significantly higher for those reporting reduced HIV care.

Positive impacts of the pandemic were rarely reported, but Leyva-Moral et al. (2023) reported that some of their participants in Spain had used the pandemic as an opportunity to adopt better self-care and healthier lifestyle habits, such as getting up early, exercising, and finding time for hobbies such as reading.

#### Coping Strategies

Few studies explored sex workers’ ways of coping, but participants across a small number of studies described several strategies of positive coping. One way of coping with the negative impacts of the pandemic was humor: two studies which explored sex workers’ social media posts found that they frequently used jokes to resist the seriousness of COVID-19 (Callander et al., 2020a) and used sarcasm, satire and irony as a way of coping (Al-Rawi & Zemenchik, 2022). Couto et al.’s (2022) study from Brazil revealed a number of positive coping strategies, including focusing on the problem, reframing and regulating emotions and “changing the meanings of the pandemic” by using the time to start new hobbies and focus on positive thinking, and drawing strength from support networks and social support. Leyva-Moral et al. (2023) noted some similar coping strategies in Spain, such as positive thinking (maintaining hope about returning to normality) and social support via phone and video calls with loved ones. Religiosity was another way of coping: Couto et al. (2022) describe how participants in Brazil searched for religion and spirituality during the pandemic, and Singer et al. (2022) describe how God and religion provided “guidance and affirmation” for sex workers in the USA during the pandemic, providing them with a foundation of support. Singer et al. (2022) also describe the use of prayer as a way of coping, with participants in this study highlighting how praying helped them to maintain psychological well-being and peacefulness. As discussed in the previous sub-section (“Impact of COVID-19 on well-being and mental health”) some sex workers increased their use of alcohol and/or drugs as a negative coping strategy (Callander et al., 2022; Couto et al., 2022; Lahav-Raz et al., 2021; Nyabeze et al., 2022; Wang et al., 2022).

### Access to Support

This theme relates to the ability of sex workers to access financial support, outreach programs and social support. Across many countries worldwide, sex workers reported being unable to access the various financial schemes implemented by governments due to their sex worker status. Migrants, Indigenous sex workers, and people of color were often less likely to be able to access financial support. The inability to access government support left sex workers feeling abandoned and excluded. Nonprofit organizations were more likely to provide support for sex workers (in terms of money, food, education and social support), but participants who offered nonprofit outreach struggled to provide support due to inconsistent funding, inability to reach sex workers due to social restrictions, and lack of protective equipment. Sex worker communities themselves were described in some studies as valuable sources of support, whereas other studies found that the pandemic caused conflict, tension and competition between sex workers.

#### Governmental Financial Support

Participants in many studies, across various countries and continents, reported being unable to access the socioeconomic protection measures (i.e., emergency financial aid) implemented by their governments (Aantjes et al., 2023; Benoit & Unsworth, 2022; Brouwers & Herrmann, 2020; Cabras & Ingrasci, 2022; Callander et al., 2022; Couto et al., 2022; Cubides Kovacsics et al., 2023; Fedorkó et al., 2022; Gonzalez & Garrido, 2022; Lahav-Raz et al., 2021; Laikram & Pathak, 2021; Mlambo & Masuku, 2022; Moura et al., 2022; Pearson et al., 2022; Pereira, 2021; Tan et al., 2021a).

Reasons for not being able to access financial support from the government included being non-status migrant workers (Benoit & Unsworth, 2022; Cubides Kovacsics et al., 2023; Fedorkó et al., 2022); not having paid taxes (Benoit & Unsworth, 2022; Callander et al., 2022; Cubides Kovacsics et al., 2023); living with relatives who had incomes (Gonzalez & Garrido, 2022); lacking the resources to apply for supports (Pearson et al., 2022); language barriers (Benoit & Unsworth, 2022; Cubides Kovacsics et al., 2023); having no bank account (Pearson et al., 2022); having no social insurance number (Benoit & Unsworth, 2022); not wanting to ‘out’ themselves as sex workers (Tümpel & Cardone, 2022); and having no documentable income (Benoit & Unsworth, 2022; Callander et al., 2022; Tan et al., 2021a). Many of these barriers were particularly relevant to migrant sex workers.

The percentages of participants able to access support varied—0% (Aantjes et al., 2023—Mozambique); 37% (Couto et al., 2022—Brazil); 56% (Tan et al., 2021a—Singapore); and 59% (Callander et al., 2022—USA). The percentage of participants able to access financial support also varied across subgroups within one study: Pearson et al. (2022) found that in Canada, 43% Indigenous and 46% women of color accessed financial support during the pandemic compared to 57% of those who were not people of color.

Pearson et al. (2022) found that some sex workers were significantly more likely to access COVID emergency income supports, namely those who used non-injection drugs, those with unstable housing, and those who had ever been incarcerated. Conversely, Indigenous sex workers were significantly less likely to access such income supports. The same study also found several factors not significantly associated with accessing COVID emergency income support, namely being a sexual minority; being transgender; previous access to sex work outreach programs; engagement with community organizations; and the primary place in which sex workers met with clients.

Governments were seen as being unresponsive of sex workers’ needs, leaving them feeling abandoned and invisible (Gonzalez & Garrido, 2022; Mlambo & Masuku, 2022; Moura et al., 2022). Many reported feeling systematically excluded from support measures and questioned the fairness of emergency relief funds (Mlambo & Masuku, 2022—South Africa); the feelings of exclusion led to anger and hurt (Benoit & Unsworth, 2022—Canada). Sex workers in Spain described how in order to access certain grants they would have to give up sex work, declare themselves victims of exploitation, or initiate hypervigilance procedures into their private lives including the care of their children (Gonzalez & Garrido, 2022). Those who could access grants in Brazil felt the grants were not adequate for reimbursing lost income or covering bills (Mlambo & Masuku, 2022).

#### Support from Nonprofit Organizations

Participants across most studies described receiving support, in some form, from NGOs, charities, allied groups and organizations set up specifically for sex workers. The types of support received included provision of necessary supplies, such as food baskets (Aantjes et al., 2023; Cabras & Ingrasci, 2022; Moura et al., 2022); supplies of ART (Aantjes et al., 2023); personal protective equipment (Benoit & Unsworth, 2022); cleaning and disinfectant products (Benoit & Unsworth, 2022); safe sex and harm reduction necessities (Benoit & Unsworth, 2022; Callander et al., 2022); and monetary support (Benoit & Unsworth, 2022; Dziuban et al., 2021; Fedorkó et al., 2022; Moura et al., 2022). Support was also provided in the form of education, such as WhatsApp courses on COVID-19 prevention (Gonzalez & Garrido, 2022) and practical help, such as multilingual translation of COVID guidelines; online workshops about working on the Internet; education about mental health and well-being; help with negotiation with landlords to prevent evictions and providing emergency accommodation; arranging for return journeys to countries of origin; legal aid; and helping sex workers obtain legal documentation, state benefits and health insurance (Fedorkó et al., 2022; Moura et al., 2022).

Overall participants who received supplies from nonprofit organizations reported being grateful for the help (Benoit & Unsworth, 2022; Dziuban et al., 2021). However, some reported reduced support from such organizations due to lockdown (Burgos & Del Pino, 2021—Spain); in the same study, few were aware of NGOs and their work in the first place. In the absence of outreach workers, many participants in Mozambique did not know how to obtain legal protection (Aantjes et al., 2023).

#### Views of Outreach and Support Organizations

Several studies included participants from nonprofit organizations which supported sex workers. They reported barriers to providing support, such as lack of consistent funding (Benoit & Unsworth, 2022; Lahav-Raz et al., 2021); being forced to close if they were defined as nonessential (Lahav-Raz et al., 2021); difficulties reaching some sex workers who were hiding from all service providers out of fear of being reported for violating COVID-19 regulations (Fedorkó et al., 2022); and lack of protective equipment despite working with at-risk populations (Lahav-Raz et al., 2021).

Their work during the pandemic consumed resources and was not seen as sustainable (Callander et al., 2022—USA); workers in Poland who provided financial support to sex workers via crowd-funding acknowledged that their help was a ‘sticking plaster’ and that systemic support was needed (Dziuban et al., 2021).

Support and outreach workers shifted their services online, but the lack of in-person groups and appointments was reported to reduce community spirit in Canada (Benoit & Unsworth, 2022). In Israel, the lack of in-person outreach also left sex workers struggling with bureaucratic barriers to accessing support, such as filling in forms and the transition to telephone/online support blurred professional boundaries, affecting the work/home balance of support workers (Lahav-Raz et al., 2021). Use of personal protective equipment in face-to-face meetings with sex workers also hindered support; Lahav-Raz et al.’s (2021) participants in Israel described how the use of face masks affected therapeutic relationships as clients felt uncomfortable talking to workers when they could not see their facial expressions.

One study in Poland found that being able to help sex workers during a time of crisis, helped outreach workers to feel empowered and gave them a sense of agency during a time of uncertainty (Dziuban et al., 2021), while another study in Israel found that lacking the resources to provide as much support as they wanted to negatively affected the support workers’ self-perception and self-worth (Lahav-Raz et al., 2021). There were also positive effects of the pandemic for workers of such organizations, who recognized their own strengths such as flexibility and ability to adapt to new ways of working; they also felt their standing in the community was strengthened, they made new collaborations, and they were better-organized (Lahav-Raz et al., 2021).

#### Social Support

Many sex workers described having very little social support during the pandemic (Burgos & Del Pino, 2021; Chakrapani et al., 2022; Mavhandu-Mudzusi & Moyo, 2022; Moura et al., 2022; Pereira, 2021; Wirawan et al., 2022). Some lacked support even pre-pandemic as they were migrants without support networks in the countries they worked in, often with colleagues their only potential source of support (Burgos & Del Pino, 2021—Spain). Others lacked social networks as they isolated themselves for fear of their commercial activity being found out; for example, Moura et al.’s (2022) participants in Portugal described having no support in adapting to home-schooling their children as they deliberately avoided developing relationships with teachers or other parents out of fear of stigmatization.

In the USA, those who did have social support found that it helped them to access financial resources, health knowledge and access to health services (Callander et al., 2022). In particular, the sex worker community was frequently cited as a valuable source of support, allowing workers to share stories and warnings about clients (Hassan et al., 2023—Kenya) and providing a sense of solidarity and being cared for (Dziuban et al., 2021—Poland). In the latter study, sex workers looked out for each other by prioritizing their colleagues when it came to receiving assistance from outreach organizations.

However, the sex work community was not always a positive force: many participants described tensions between workers. For example, Callander et al.’s (2022) participants in the USA reported arguments between workers directed at those who continued to offer in-person services and a hierarchy of power among sex workers that reinforced racism and classism; the more affluent workers who were able to discontinue in-person services were perceived as enacting stigma against those who could not. Those who remained on the street competed over clients and space which resulted in verbal and physical fights, a lack of unity and reduced peer support (Aantjes et al., 2023; Laikram & Pathak, 2021). Conflicts and competition with colleagues were also reported by Couto et al. (2022) and Kahambing (2021), although the latter study (whose participants were massage therapists who also engaged in sex work) also described support between colleagues, suggesting they had empathy for each other and served as “shoulders to cry on” for each other.

### Access to Health Care

A number of studies explored sex workers’ access to health care during the pandemic, including health care in general, COVID-19 care, sexual and reproductive care, and HIV care. Although findings relating to the ability to access care were mixed, barriers were identified including preexisting stigma, COVID-19 restrictions, lack of transport, and fear of catching COVID-19 or experiencing harassment for leaving their homes.

#### Health Care in General

Several studies found that participants reported no, or few, problems with accessing health care. For example, 83% of Mantell et al.’s (2021) participants in Kenya reported the pandemic did not affect their ability to obtain health care as did 84% of Tan et al.’s (2021a) participants in Singapore. Pereira’s (2021) participants in Portugal reported no difficulty accessing health care, but described how they had to become more efficient at managing their time to fit in appointments because they were much more limited than usual.

However, other studies reported much higher rates of healthcare access difficulties (e.g., Leyva-Moral et al., 2023—Spain). Approximately 35% of Museva et al.’s (2021) participants in Zimbabwe reported limited access to medication. In Tan et al.’s (2021b) study from Singapore, 68% of participants felt the pandemic led to poorer access to health care.

Barriers to accessing health care during the pandemic included not wanting to draw attention to their place of work or their migrant status (Burgos & Del Pino, 2021); preexisting stigma and fear of being thought of as sex trafficking victims (Callander et al., 2022); inability to travel to healthcare sites (Mantell et al., 2021); fear and distrust of mainstream healthcare services (Stevens et al., 2021); language barriers meaning they could not understand COVID-19 protection guidelines (Fedorkó et al., 2022); restrictions in movement due to COVID-19 (Museva et al., 2021); lack of health insurance (Callander et al., 2022) and fear of receiving large bills for health care (Fedorkó et al., 2022); long waiting lists (Leyva-Moral et al., 2023); immigrant status (Fedorkó et al., 2022; Leyva-Moral et al., 2023); and being stranded outside of their usual cities due to COVID-19 regulations and not knowing where to access health care (Gichuna et al., 2020).

Folayan et al. (2022) found that women in Nigeria who sold sex had significantly higher odds of reporting limited access to tuberculosis services than other population groups. Within the sex worker community in Singapore, reduced access to medical and healthcare services was associated with being transgender (Tan et al., 2021a).

#### COVID-19 Care

While some sex workers reported receiving COVID-19 supplies such as masks and sanitizers (Mlambo & Masuku, 2022—South Africa), others felt they would have difficulty getting COVID-19 tests or treatment (Gbagbo, 2020—Ghana) and reported having no health checks for those symptomatic of COVID-19 (Burgos & Del Pino, 2021—Spain). In Chiang et al.’s (2022) study from Brazil, 83% of participants had not received a COVID-19 vaccine, despite the majority of them meeting vaccination criteria and many having preexisting health conditions.

#### Sexual and Reproductive Care

In Wirawan et al.’s (2022) Indonesian study, 83% of participants felt there was either no change in access to condoms, or it became easier in the pandemic to access them; similarly, Jones et al.’s (2022) participants in Ethiopia referred to strong links to sexual/reproductive health programs and counselors where they could get regular HIV/STI tests and condoms. However, many of Museva et al.’s (2021) participants in Zimbabwe reported losing access to contraception, and many of Gichuna et al.’s (2020) participants in Kenya were missing reproductive health commodities such as family planning options and pregnancy tests, reporting that they felt reproductive health was forgotten amid the pandemic. Economic challenges also undermined access to reproductive health care, with lack of money for transport making it difficult to get to appointments (Gichuna et al., 2020—Kenya). Nyabeze et al.’s (2022) participants in Zimbabwe reported no longer being able to afford condoms or access them because the brothels and night clubs where they used to obtain them had shut, while Leyva-Moral et al. (2023) found that access to condoms in Spain was limited due to closures of NGOs.

Santos et al. (2021, 2022) found that sex workers globally were significantly more likely than populations not considered “vulnerable” to have no or reduced access to condoms or lubricants and Folayan et al. (2022) found that women in Nigeria who sold sex had higher odds than other populations of reporting limited access to sexual and reproductive health services.

#### HIV Care

Some studies revealed little change in access to HIV care for sex workers during the pandemic. For example, in Zimbabwe and South Africa, respectively, PrEP uptake was found to remain consistent or even improve during the early months of the pandemic in two studies (Matambanadzo et al., 2021; Rao et al., 2022).

However, in other studies participants reported problems with accessing PrEP (Gichuna et al., 2020; Moyo et al., 2022); missing HIV care visits (Wang et al., 2022); and difficulties accessing refills of HIV medications such as ARVs (Gichuna et al., 2020; Moyo et al., 2022; Pollard et al., 2021; Santos et al., 2021; Wang et al., 2022). Participants in Zimbabwe also described a compromised quality of care, with cessation of cervical screening and viral load monitoring; lack of time in appointments to share their concerns; and reduced interactions with healthcare staff (Moyo et al., 2022). Magnani et al. (2022) found that by July 2020, mobile HIV testing services in Indonesia only rebounded to about one-fifth of their February 2020 level but the starting of treatment and retention of ART largely recovered to pre-pandemic levels.

Barriers to HIV prevention and treatment included medication shortages (Matambanadzo et al., 2021; Pollard et al., 2021); forfeiting clinic visits in order to work during the day, due to COVID-19 curfews (Gichuna et al., 2020); not wanting to disclose their private information (such as HIV status) in order to enter healthcare facilities (Moyo et al., 2022; Tran et al., 2022); confidentiality concerns about getting HIV medicines or testing at home (Pollard et al., 2021); the inconvenience of long waiting times (Gichuna et al., 2020); not wanting to stand outside for COVID screening where they risked being seen by clients (Moyo et al., 2022); no ability to obtain authorization letters to allow them to travel to treatment centers (Moyo et al., 2022); lack of money for transport to clinics (Moyo et al., 2022); closure of clinics (Leyva-Moral et al., 2023); avoiding traveling to clinics for fear of catching COVID-19 or experiencing police harassment for leaving the house (Pollard et al., 2021); no access to smartphones or computers for telemedicine (Pollard et al., 2021); and not knowing where to access care (Gichuna et al., 2020). In a study from Zimbabwe, sex workers who struggled with food insecurity during the pandemic were also at heightened risk of not adhering to or discontinuing HIV medication as it was not easy to take the medication on an empty stomach (Moyo et al., 2022).

Santos et al., (2021, 2022) found that sex workers across the world were significantly more likely than populations not considered ‘vulnerable’ to have problems accessing or refilling ART medication, and were more likely to report not being able to see an HIV treatment provider. Additionally, Folayan et al. (2022) found that women in Nigeria who sold sex, along with transgender women, had significantly higher odds of reporting limited access to HIV services during the pandemic than all other “vulnerable” groups (including people with disabilities, people on the move, people who engaged in “transactional sex,” and illegal drug users). Within the sex worker population in the Dominican Republic, Wang et al. (2022) found that participants with and without reduced HIV care did not differ in any sociodemographic characteristics, but reduced HIV care was significantly higher among sex workers who experienced increased verbal and emotional abuse from partners during the pandemic; sex workers with higher financial concerns; and sex workers with greater COVID-19 mental health problems.

### Impact of COVID-19 on Research with Sex Workers

The final theme relates to the research itself, and the barriers to including sex workers in academic research.

Several authors pointed out the difficulties of making contact with sex workers during the pandemic for purposes of research and assessment. For example, Laikram and Pathak’s participants, who were government officials in Thailand, described how it was impossible to properly ascertain the full monetary damage of the pandemic or carry out loss assessment among sex workers as only “visible” ones would be included; as this review has shown, many became (even more) invisible during the pandemic for a multitude of reasons. In a study from Kenya, Gichuna et al. (2020) commented on the difficulties associated with trying to carry out telephone interviews with sex workers during the pandemic’s lockdown measures: phones were often shared, not charged, or had no data, and it was also difficult to discuss sex work activities when living in communal settings. Shankar et al. (2022) also noted the difficulty of accessing sex workers in India post-lockdown as many migrated to their native towns/villages during the pandemic and were therefore inaccessible. Silva and dos Santos Câmara (2020) conclude that novel approaches need to be considered in order to retain contact with vulnerable research participants such as those in the sex work community.

## Discussion

This review of 63 studies revealed the impact of COVID-19 on the lives of sex workers, at structural, social and individual levels. Firstly, we noted that almost every included study provided data pertaining to reduction in work and consequent reduction in income. This is not surprising, given the closure of venues where sex would have taken place, the restrictions on mobility, and the fact that by its nature sex work involves intimate physical contact and governments across the world were warning their citizens to physically distance from others. The reduction in clients and venues for sex work left sex workers needing to work more than ever, as many were struggling to pay for rent, bills and food. As a result, sex workers in nearly all studies reported feeling they had no option but to continue working during the pandemic. Since there were fewer potential clients, and greater competition among sex workers, many felt pressured to lower their prices or engage in risky behaviors such as unprotected sex which they would not usually have agreed to. They also faced increased abuse and violence from clients as well as from police and security officers enforcing the COVID-19 regulations.

Due to the nature of their work, social distancing was not possible—sex workers have close physical contact for prolonged periods of time so are at high risk of catching COVID-19. Indeed, participants in many studies expressed fear of infection and infecting their families. While many used protective measures, their work still involved a high level of physical contact, and many clients refused to wear masks. The combination of reduced finances, isolation from others, and fear of infection exacerbated existing mental health problems and lowered the psychological and emotional well-being of sex workers. This is not surprising as studies of numerous populations across the world have reported the same effects after pandemics (Brooks et al., 2020).

Many sex workers shifted to offering online services during the pandemic; this is not unsurprising given that moving to digital platforms has been reported by other freelance workers (Brooks & Patel, 2022). However, this is problematic for two reasons. Firstly, those who do not have access to computers, smartphones or the Internet, or lack the skills to use them for their work, are excluded from the opportunity in what Baker et al. (2020) refer to as the “digital divide” between those in society with access to computers and those without. Secondly, those that can access digital platforms may be placing themselves at risk: there have been suggestions that online commercial sex is actually riskier than street-based commercial sex, with workers reporting persistent or unwanted contact through email or social media (Campbell et al., 2019). Various strategies for enhancing sex workers’ safety online have been previously found to be useful, such as electronic lists of abusive clients being made available to sex workers via outreach organizations; closed Facebook groups, WhatsApp chats and sex worker forums for sharing information and warnings; negotiating services and fees via email before meeting clients; and reporting abusive behavior to Web site administrators (Bernier et al., 2021).

Our review revealed that for sex workers across the world, COVID-19 impeded access to a diverse range of health services, including COVID-19 services, sexual and reproductive services and HIV care. While this finding is not unique to sex workers (Moynihan et al., 2021), several participants described how access was impeded by sex work stigma preceding the pandemic. Sex workers have long reported mainstream general practice and mental health services being largely inaccessible to them, for example due to appointment systems not being appropriate to their needs, difficulties registering with general practitioner (GP) surgeries, inability to pay for the cost of telephone calls to GP surgeries and fear or experience of stigma or judgment from staff (Mastrocola et al., 2015; Potter et al., 2022). Sex workers have also been reported to fear seeking health care, pre-pandemic, due to the stigma and discrimination they are exposed to in their communities and fear of being exposed to the authorities (Taylor-Robinson et al., 2021).

Several studies raised concerns about gaining access to sex workers for research purposes during a public health crisis such as the COVID-19 pandemic. Sex workers are already likely to be a hard-to-reach population, given the prejudice and stigma surrounding the profession as well as the criminality of sex work in most countries. Research also suggests those engaged in transactional sex are at increased risk of loss to follow-up in research studies (Kabarambi et al., 2022). Given that much of the research carried out during the pandemic had to be done via telephone or online due to social distancing measures, sex workers without access to technology would have been unlikely to take part—meaning there may be a hidden population of sex workers whose pandemic experiences have not been explored. In other words, there are potentially many sex workers in even more dire circumstances than the more “visible” ones who took part in research studies; therefore, researchers may not truly know the extent of the impact the COVID-19 pandemic has had and continues to have on the most vulnerable members of this population.

It is important to note that, while the COVID-19 pandemic certainly appears to have exacerbated existing problems for sex workers, their struggle for survival is not unique to the pandemic. Even before the crisis, many were extremely vulnerable (Dziuban et al., 2021); the COVID-19 is likely to have intensified vulnerabilities which already existed within the sex worker community (Azam et al., 2021). We found a small amount of literature on the ways in which sex workers coped with the pandemic, with some studies suggesting positive thinking, using humor, drawing on social support when this was available, and religiosity might be protective of sex workers’ well-being. However, only a small number of studies explored coping strategies, and future research should aim to explore the different ways in which sex workers coped emotionally during the pandemic and which ways of coping were most helpful.

The literature reflects resources which could help mitigate the loss of work and income experienced by sex workers: for example, technological skills and access to smartphones or computers; non-sex work employment; health knowledge; financial support; and social support. Therefore, it would be useful for outreach programs to aim to mitigate loss of income by incorporating these resources. For example, such programs should aim to develop sex workers’ technology-related skills; provide smartphones or computers; help sex workers identify and access potential non-sex-related employment opportunities; provide accessible health information including pandemic-related guidelines; offer financial support; and offer a safe place for sex workers to be supported by outreach workers or facilitate the access of social support within communities. However, we acknowledge that the studies included in this review which explored the experiences of outreach workers suggest that it is difficult for programs to provide these resources due to inconsistent funding and support. As a result, many sex workers—and in particular the most vulnerable of this already vulnerable group—do not have access to these resources. This is why many of the negative effects experienced during the pandemic (e.g., loss of work, loss of income, risky behaviors, poor mental health, and loss of access to health care and support) appear to be particularly prominent at intersections of socioeconomic status, immigration status, race and gender identity. Most of the studies in our review which statistically examined the effects of the pandemic across different subgroups of sex workers found that particular groups of sex workers (such as people of color, transgender individuals, migrants, mothers, and sex workers with low socioeconomic status or low education) faced intersectional disparities in relation to the impact of COVID-19 and their access to resources. Overall, the impact of COVID-19 on sex workers has been largely negative, with certain subgroups disproportionately affected: as Shankar et al. (2022) point out, sex workers are not a heterogeneous population and a closer examination of how the pandemic has affected diverse groups within the population is needed. Researchers in the field should therefore be sensitive to the importance of intersectionality in research with sex workers, and should consider how sex work intersects with other oppressions (such as race, gender identity, socioeconomic status, and migrant status).

### Implications for Research and Practice

In terms of gaps in the literature, we found very few longitudinal studies and in fact few considering later effects of the pandemic, with the majority collecting their data in 2020. Given the timing of this review (December 2022), it is not surprising that the majority of studies published thus far are based on data gathered fairly early in the pandemic. So while this review provides an important snapshot of the state of the literature 3 years into the pandemic, we are hopeful that more studies in the future will examine the long-term effects of the pandemic on sex workers. We also, perhaps surprisingly, found relatively few studies considering the mental health of this population: the majority focused on economic impacts of the pandemic and access to health care, overlooking the impact on well-being. While some studies did examine mental health and well-being, none quantitatively assessed mental health using standardized measures of mental health disorders. Based on what is already known about the psychological impact of pandemics (Brooks et al., 2020), we expected to find more studies assessing levels of depression, anxiety and post-traumatic stress in this population. Additionally, we found few studies assessing the number of sex workers who actually caught COVID-19, and the potential long-lasting impact on their physical, financial, or emotional well-being. We also hope that more research in the future will explore gender identity in relation to the effects of the COVID-19 pandemic on sex workers. Many studies focused on female sex workers, overlooking male sex workers and often failing to report on whether their study samples were cisgender, transgender, or a combination. Finally, we found very few studies considering resilience, post-traumatic growth, or any other opportunities or positive impacts experienced by sex workers during the pandemic. It may be the case that few positive impacts were felt; however, it may also be the case that this simply has not yet been explored. In order to better understand how to support the mental health of the sex worker community if another public health crisis were to occur, it would be useful to understand factors associated with growth, individual and group resilience and positive coping in this population.

The findings of this review also have numerous implications for policy and practice. First and foremost, our results show that sex workers require financial support during a public health crisis such as COVID-19 which substantially reduces their ability to work or in some cases prohibits them from working completely. Regardless of the morality of their work, it is vital to improve sex workers’ access to government-based financial support and policies should be put in place to ensure this population is not forgotten. Similarly, it must be ensured that access to social and health care for this group is also not forgotten. The “leave no one behind” concept in the context of public health was emphasized in the 2030 Sustainable Development Goals (United Nations, 2016), which urged for universal access to health care and education; the end of discrimination and exclusion; and reduction in inequalities that leave certain groups more vulnerable than others. Reaching the goal of leaving no one behind during a prolonged and severe crisis such as the COVID-19 pandemic is understandably complex and unfortunately our results suggest that sex workers *have* felt “left behind,” with many reporting feeling abandoned, excluded, and forgotten. It would be helpful for public health authorities to work collaboratively with representatives of sex work organizations (including sex workers themselves) when developing policies and interventions, so that the community itself is given a voice in decision-making.

Sex workers also need provisions of both supplies and education during a prolonged public health crisis. For example, our review suggests that they would have benefited from free and accessible provision of food baskets; supplies such as sanitizer, masks, PPE and access to contact tracing; COVID-19 tests; contraceptives; educational resources about safer sex in the context of COVID-19; and informational resources providing education about COVID-19 and how to minimize the risk of catching and spreading the virus. While we found many studies detailing how sex worker-led nonprofit organizations took steps to provide these, many reported no consistent funding for these organizations meaning they struggled to help everyone who needed to be helped. It is essential that community-based organizations designed to support sex workers should have sufficient funding and resources to support the sex worker population during a prolonged crisis.

While we found very little literature examining COVID-19 vaccination intentions, and uptake, in sex workers; the evidence we did find suggested potentially low vaccination rates. Given that sex workers who continue to work during public health crises are particularly at risk of infection and in need of vaccination, we suggest that intervention is needed to improve uptake. Vaghela et al. (2021) propose that vaccine uptake could be improved in this population by including sex workers in vaccine priority lists; authorities working with sex worker organizations to develop effective communication strategies to combat apprehension around vaccines; and use of mobile or pop-up clinics which do not require proof of identity in order to receive vaccines.

Public health authorities should also consider how to ensure that information and guidelines are accessible and relevant to sex workers. This could be done by developing public health messaging tailored toward sex workers specifically—for example, Karamouzian et al. (2020) suggest that public health messages regarding self-isolation and social distancing should be modified for marginalized groups such as sex workers. Additionally, given that many sex workers are migrants who are not always fluent in the language of the countries they are based in, it is important that public health information is available in all languages and accessible to all, even those without access to technology. Again, sex worker organizations could help with ensuring that sex workers have access to public health guidelines.

Workshops led by and for sex workers to address both physical and mental health concerns during a public health crisis may also be useful (van Ravenswaaij et al., 2021). Interventions using peer design and peer delivery have previously been found to be effective in improving health and determinants of health in sex workers (Johnson et al., 2022), and approaches based on education and empowerment have also been found to be successful (Johnson et al., 2022; van Ravenswaaij et al., 2021) therefore interventions should focus on both empowerment and inclusion of sex workers’ voices. Lam (2020) urges that instead of viewing sex workers as needing to be “rescued” (which is disempowering and furthers the oppression they experience) interventions should recognize and respect the agency of sex workers and their rights. It is important for the voices of marginalized groups such as sex workers to be heard and thus important for them to be involved in decisions, policymaking, and interventions.

### Limitations

Perhaps the most important limitation of our own review process is the fact that literature searches were restricted to English-language papers due to our own lack of resources for translation. Given that sex work is a global phenomenon, it is important that studies published in other languages are not overlooked. We recommend that future reviews of this topic should not restrict to any particular language and should employ translation services to ensure that studies in all languages are included. Secondly, unpublished and gray literature were considered outside of the scope of this review due to our focus on literature that was peer-reviewed (or, at least, editor-reviewed): future reviews might consider including gray literature, reports, unpublished dissertations and preprints for a broader view of the literature. Using a greater number of search terms, searching additional databases and hand-searching key journals may have yielded more results and so we would recommend that future reviews expand on our search and include more than the six databases we searched. Another limitation of the current review is the difficulty to make transferable conclusions given that the results are from studies across the world with very different sociocultural contexts. Future syntheses of literature may focus on similar countries, in order to establish culturally specific conclusions, but we hope this review provides a good basis for doing this. Finally, as this was a scoping review (rather than a systematic review) we did not carry out any formal appraisal of the quality of included studies. This is a limitation and we recommend that any researchers who expand on our work to carry out further reviews should include an element of formal quality assessment in order to ascertain whether the findings of particular studies should perhaps be given more or less weight in the discussion of results.

### Conclusion

The COVID-19 pandemic has had a substantial effect on the livelihoods of sex workers across the world. Social distancing measures have led to reduced demand for sex workers, which has led to a loss of clients and income, leaving many struggling with housing and food insecurity. The need for income has led many sex workers to engage in risky practices such as unsafe sex and not vetting potential clients beforehand, associated with an increase in workplace violence and aggression directed toward them. Many felt at risk of catching COVID-19 but pressured to continue working anyway. The combination of reduced income, fear of infection and isolation from sources of social support is reported to have negatively affected mental health. Sex workers have also faced difficulties accessing both financial support and health care during the pandemic due to a combination of stigmatization, restrictions on mobility due to the pandemic, and issues relating to sex worker status and often migrant status. Further research is needed to understand the long-term impact the pandemic could have on this vulnerable population. In order to ensure sex workers are not left behind in crisis response, it is important to provide support and resources to peer-led sex worker organizations.

## Data Availability

Not applicable—no new data were generated for this review.

## Appendix 1: Search Strategy

1. “sex work*”
2. prostitut*
3. sex industr*
4. brothel*
5. escort*
6. 1 OR 2 OR 3 OR 4 OR 5
7. COVID*
8. coronavirus
9. sars-cov-2
10. lockdown*
11. pandemic*
12. 8 OR 9 OR 10 OR 11
13. 6 AND 12

Date limit: December 2019–current
